# Immunogenic particles with a broad antigenic spectrum stimulate cytolytic T cells and offer increased protection against EBV infection *ex vivo* and in mice

**DOI:** 10.1371/journal.ppat.1007464

**Published:** 2018-12-06

**Authors:** Dwain G. van Zyl, Ming-Han Tsai, Anatoliy Shumilov, Viktor Schneidt, Rémy Poirey, Bettina Schlehe, Herbert Fluhr, Josef Mautner, Henri-Jacques Delecluse

**Affiliations:** 1 German Cancer Research Center (DKFZ) Unit F100, Heidelberg, Germany; 2 Faculty of Biosciences, Heidelberg University, Heidelberg, Germany; 3 Institut National de la Santé et de la Recherche Médicale (INSERM) Unit U1074, Heidelberg, Germany; 4 German Center for Infection Research (DZIF), Braunschweig, Germany; 5 Frauenklinik, University Hospital Heidelberg, Heidelberg, Germany; 6 Children’s Hospital, Technische Universität München, & Helmholtz Zentrum München, Munich, Germany; QIMR Berghofer Medical Research Institute, AUSTRALIA

## Abstract

The ubiquitous Epstein-Barr virus (EBV) is the primary cause of infectious mononucleosis and is etiologically linked to the development of several malignancies and autoimmune diseases. EBV has a multifaceted life cycle that comprises virus lytic replication and latency programs. Considering EBV infection holistically, we rationalized that prophylactic EBV vaccines should ideally prime the immune system against lytic and latent proteins. To this end, we generated highly immunogenic particles that contain antigens from both these cycles. In addition to stimulating EBV-specific T cells that recognize lytic or latent proteins, we show that the immunogenic particles enable the *ex vivo* expansion of cytolytic EBV-specific T cells that efficiently control EBV-infected B cells, preventing their outgrowth. Lastly, we show that immunogenic particles containing the latent protein EBNA1 afford significant protection against wild-type EBV in a humanized mouse model. Vaccines that include antigens which predominate throughout the EBV life cycle are likely to enhance their ability to protect against EBV infection.

## Author summary

Human herpesviruses are tremendously successful pathogens that establish lifelong infection in a substantial proportion of the population. The oncogenic γ-herpesvirus EBV, like other herpesviruses, expresses a plethora of open-reading frames throughout its multifaceted life cycle. We have developed a prophylactic vaccine candidate in the form of immunogenic particles that contain several EBV antigens. This is in stark contrast to the vast majority of EBV vaccines candidates that contain only one or two EBV antigens. Our immunogenic particles were shown capable of stimulating several EBV-specific T-cell clones *in vitro*. The immunogenic particles were also capable of expanding cytolytic EBV-specific T cells *ex vivo* and provided a protective benefit *in vivo* when used as a prophylactic vaccine.

## Introduction

The Epstein-Barr virus (EBV) is a γ-herpesvirus that establishes asymptomatic infection in the majority of the human population. EBV infects both B cells and epithelial cells, but it is in the former in which EBV establishes latency and persists lifelong [1]. Despite being carried asymptomatically by most individuals, the global disease burden of EBV is substantial. EBV is the primary cause of infectious mononucleosis (IM), accounts for 200,000 new cancer cases annually [2] and is linked to the development of autoimmune diseases (e.g. multiple sclerosis) [3].

Shortly after the discovery of EBV, vaccination was touted as a possible means of controlling or eliminating EBV-associated diseases [4]. Despite EBV being the first human oncogenic virus to be discovered, and in spite of several decades of EBV vaccine research, no prophylactic EBV vaccine has made it onto the market. So far, the majority of prophylactic vaccine prototypes have focused on the major viral envelope glycoprotein gp350 [5]. One study, in which soluble gp350 was used for vaccination purposes, reported a decrease in the frequency of IM in vaccinated individuals over a study period of 18 months, but vaccination did not reduce the frequency of infection with the wild type virus [6]. Long-term information on the vaccinated cohort is not available.

Herpesviruses have complex life cycles and primary infection, latency and reactivation are achieved through the expression of a large number of open-reading frames [7–9]. Considering the number of antigens that are expressed during the EBV life cycle, it is not surprising that EBV infection is controlled in healthy individuals through humoral and cellular immune responses that target a variety of lytic and latent proteins [10]. Considering the breadth of EBV-specific immune responses in healthy individuals, it is surprising that EBV vaccine prototypes have only targeted a limited set of lytic [6,11,12] or latent proteins [13].

We previously generated a vaccine candidate in the form of EBV virus-like particles (VLPs) and light particles (LPs) [14]. Deletion of EBV proteins involved in DNA packaging produced particles that were DNA-free, non-infectious and highly immunogenic. Whilst EBV VLPs/LPs are likely to contain several dozen lytic proteins (*viz*. envelope, tegument and capsid proteins) [15], they are devoid of latent proteins. To address this shortcoming, we enlarged the antigenic spectrum of VLPs/LPs to include immunodominant latent proteins. We interrogated the antigenicity of VLPs/LPs containing latent antigens *in vitro*, *ex vivo* and *in vivo*.

## Results

### Enlarging the antigenic spectrum of EBV virions to include latent proteins

**Fig 1:**
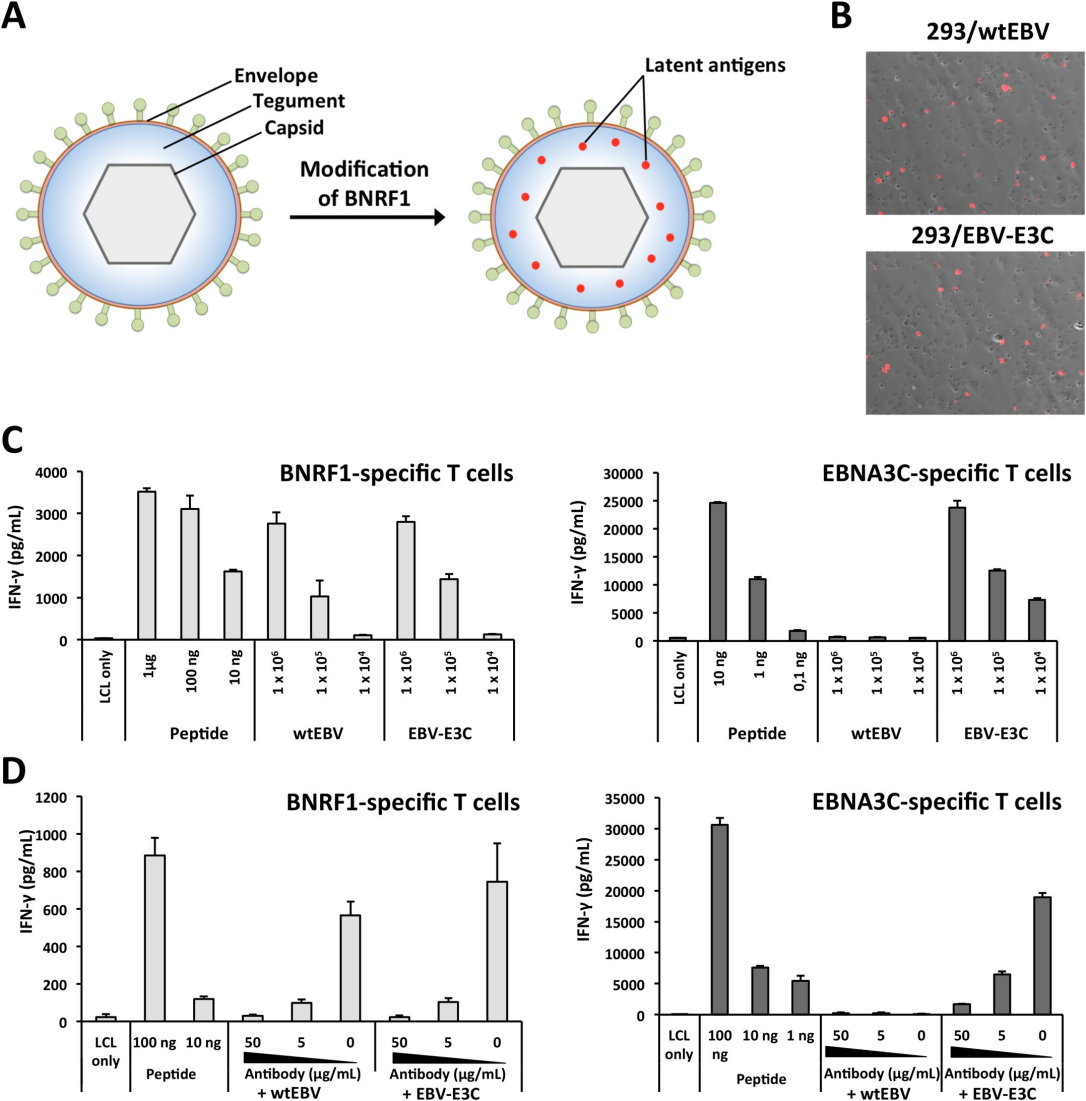
The antigenic spectrum of EBV virions is enlarged through the construction of BNRF1-latent protein gene fusions. (A) The insertion of latent protein epitopes into the major tegument protein BNRF1 enables them to be incorporated into the tegument of VLPs/LPs. (B) Modification of BNRF1 to include an antigenic fragment from EBNA3C (E3C) does not influence lytic replication in producer cells. The late lytic gene product gp350 was detected at the surface of induced 293/wtEBV and 293/EBV-E3C producer cells by staining with α-gp350 and α-mouse IgG-Cy3 antibodies. (C) EBV virions that encode a BNRF1-EBNA3C fusion protein stimulate BNRF1- and EBNA3C-specific CD4^+^ T cells. Autologous LCLs were pulsed with various amounts (1 x 10^4^ to 1 x 10^6^ genome equivalents (geq)) of wtEBV or EBV-E3C and then cocultured with CD4^+^ T cells that were specific for BNRF1 VSD (1006–1017 aa) or EBNA3C 5H11 (325–339 aa) epitopes. In parallel, LCLs were pulsed with control peptides (μg to ng quantities) prior to coculture with CD4^+^ T cells. T-cell activation was determined by measuring secreted IFN-γ by ELISA. (D) A neutralizing antibody that recognizes gp350 impairs the antigenicity of wtEBV and EBV-E3C. The neutralizing antibody 72A1 was titrated (50, 5 and 0 μg/mL) and incubated with 1 x 10^6^ geq of wtEBV and EBV-E3C. Thereafter, supernatants were used in T-cell activation assays. The data displayed in each chart represents triplicate values and error bars represent standard deviation. Furthermore, each graph is a representative experiment of at least three.

To generate immunogenic particles with an enlarged antigenic spectrum, we aimed to introduce latent protein fragments into EBV VLPs/LPs. Since BNRF1 is abundant within virions [15], we rationalized that latent antigens could be introduced into VLPs/LPs by fusing them to BNRF1 (Fig 1A). However, since wild-type EBV (wtEBV) can be accurately and sensitively quantified through qPCR [14], we first modified BNRF1 of wtEBV to test this assumption. The BNRF1 of wtEBV was modified to contain a fragment from the highly antigenic latent protein EBNA3C [16] (S1 Fig). Bacterial artificial chromosome (BAC) DNA from wtEBV was modified to contain EBNA3C and then stably introduced into 293 cells to generate a virus producer cell line (293/EBV-E3C). The integrity of the EBV-E3C BAC DNA within producer cells was confirmed with restriction analysis (S1 Fig). Transfection of the lytic transactivator BZLF1 gene into 293/EBV-E3C and 293/wtEBV yielded a similar percentage of cells that expressed the late lytic protein gp350 (Fig 1B). This indicates that modification of BNRF1 to include a latent antigen fragment does not influence lytic replication. Next, we compared the antigenicity of EBV-E3C and wtEBV viruses in T-cell activation assays (Fig 1C). Autologous lymphoblastoid cell lines (LCLs) were pulsed with the two viruses or peptide controls and then cocultured with BNRF1- [17] or EBNA3C- [18] specific CD4^+^ T cells. This confirmed that modified virions, containing a BNRF1-EBNA3C fusion protein, were able to simulate EBNA3C- and BNRF1-specific CD4^+^ T cells. Conversely, wtEBV that contained unmodified BNRF1 was only able to stimulate the BNRF1-specific CD4^+^ T cells (Fig 1C). In all cases, the dose of the virus applied correlated to the response generated by the T cells, with as little as 1 x 10^4^ virions (genome equivalents (geq)) being able to generate responses from the BNRF1- and EBNA3C-specific T cells. Importantly, BNRF1-specific CD4^+^ T cells recognized modified and unmodified EBV to the same extent. This indicates that BNRF1-latent antigen fusion proteins enlarged the antigenic spectrum of EBV without influencing the antigenicity of BNRF1. Next, we tested whether the enlarged antigenic spectrum of EBV-E3C was exclusively due to BNRF1-latent antigen fusions contained within virions. To this end, virus supernatants were pre-incubated with anti-gp350 neutralizing antibody [19] prior to being used in T-cell recognition assays (Fig 1D). This showed that the neutralizing antibody was able to abolish the antigenicity of EBV-E3C. Altogether, these results confirm that BNRF1-latent antigen fusion proteins are successfully packaged into virions and enlarge their antigenic spectrum.

### Antigenic diversification of VLPs/LPs lacking gp110

**Fig 2:**
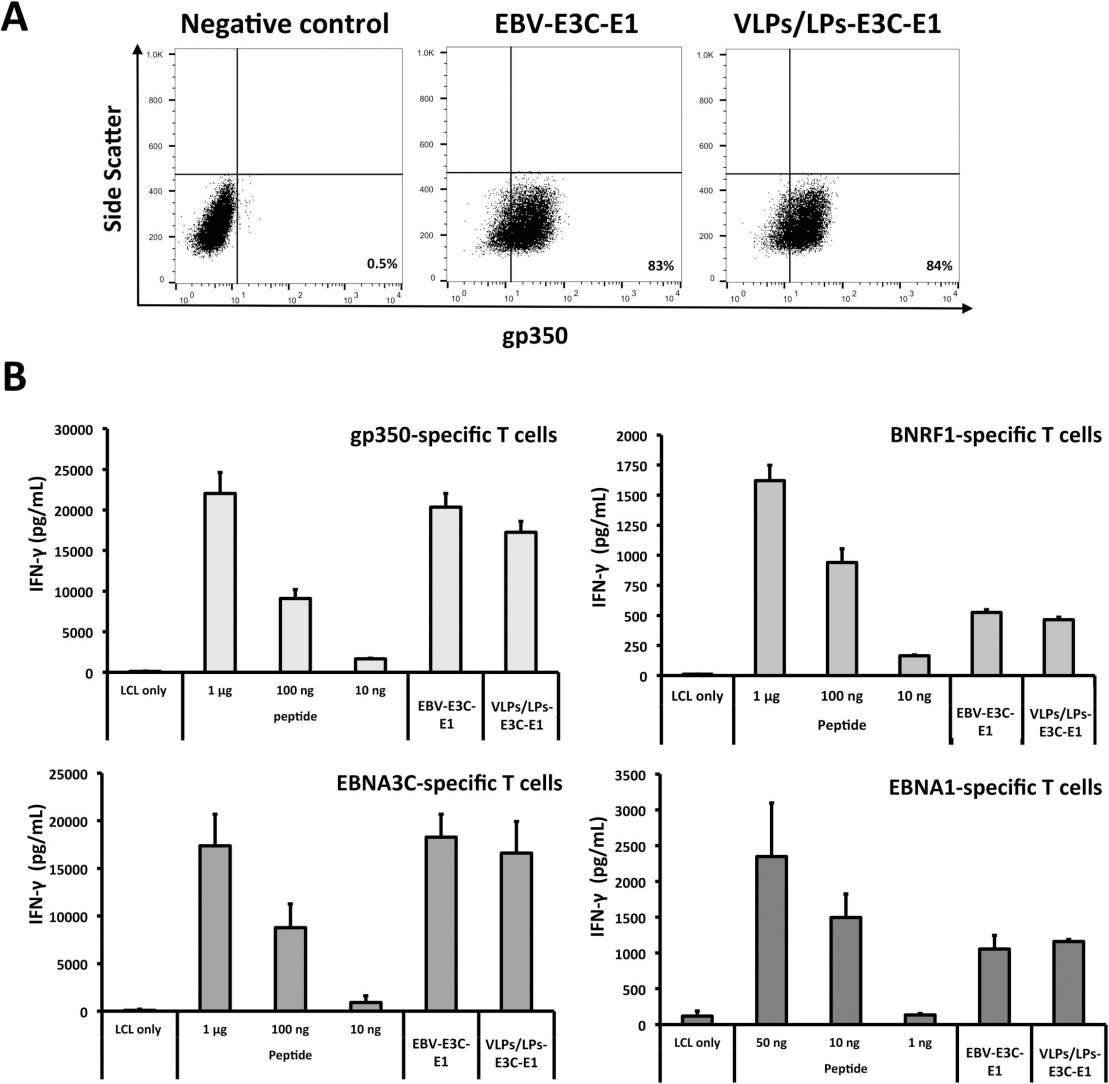
Modified VLPs/LPs that lack gp110 are antigenic and stimulate multiple EBV-specific T cells. (A) VLPs/LPs and EBV that contain EBNA3C (E3C) and EBNA1 (E1) bind to B cells to a similar degree. Equal quantities of EBV-E3-E1 (1 x 10^6^ geq) and VLPs/LPs-E3C-E1 (1 x 10^6^ particles) were incubated with Elijah B cells, stained with α-gp350 (72A1) and α-mouse IgG-Cy3 antibodies and then analysed with flow cytometry. The values displayed on plots indicate the percentage of B cells that were bound by virus or VLPs/LPs. (B) VLPs/LPs-E3C-E1 retain their antigenic character in the absence of gp110. Autologous LCLs were pulsed with control peptides, VLPs/LPs-E3C-E1 (1 x 10^6^ particles) or EBV-E3C-E1 (1 x 10^6^ geq) and cultured with T cells that were specific for gp350 1D6 (65–69 aa), BNRF1 VSD (1006–1017 aa), EBNA3C 5H11 (325–339 aa) or EBNA1 3E10 (475–489 aa) epitopes. T-cell activity was determined by quantifying IFN-γ release with ELISA. The data illustrated in the graphs are average of triplicate values and error bars represent standard deviation. Furthermore, each graph is a representative experiment of at least three.

Next, we confirmed the antigenicity of BNRF1-latent antigen fusion proteins in gp110-negative VLPs/LPs. Since gp110 has been shown to preclude viral and host membrane fusion [20], and abrogate toxicity [21], we exclusively used gp110-negative VLPs/LPs in the present study. We concurrently modified VLPs/LPs and wtEBV to encode EBNA3C and EBNA1 fragments, respectively generating 293/VLPs/LPs-E3C-E1 and 293/EBV-E3C-E1 producer cells (S2 Fig). EBNA1, like EBNA3C, is a highly immunogenic latent protein that is frequently recognized by the population [16,22]. The DNA-free VLPs/LPs-E3C-E1 were quantified using flow cytometry (S3 Fig) and then compared to an equivalent amount of EBV-E3C-E1 that was quantified with qPCR (Fig 2A). This confirmed that flow cytometry enabled the reliable quantification DNA-free VLPs/LPs. Next, VLPs/LPs-E3C-E1 were analysed in T-cell activation assays alongside EBV-E3C-E1 (Fig 2B). This showed that the VLPs/LPs, like EBV virions, were able to stimulate BNRF1- [17], gp350- [23], EBNA3C- [18] and EBNA1- [24] specific CD4^+^ T cells when they were modified to contain EBNA3C and EBNA1 fragments (Fig 2B). Furthermore, the modified VLPs/LPs stimulated the various lytic protein- and latent protein-specific T cells to the same extent as modified EBV. This confirmed that VLPs/LPs could be used as a platform to generate immunogenic particles that comprise lytic and latent antigens. Furthermore, the lack of gp110 does not negatively influence the antigenicity of the VLPs/LPs, indicating that their safety can be increased without compromising their antigenicity.

### Modified VLPs/LPs expand T cells that efficiently control recently infected B cells

**Fig 3:**
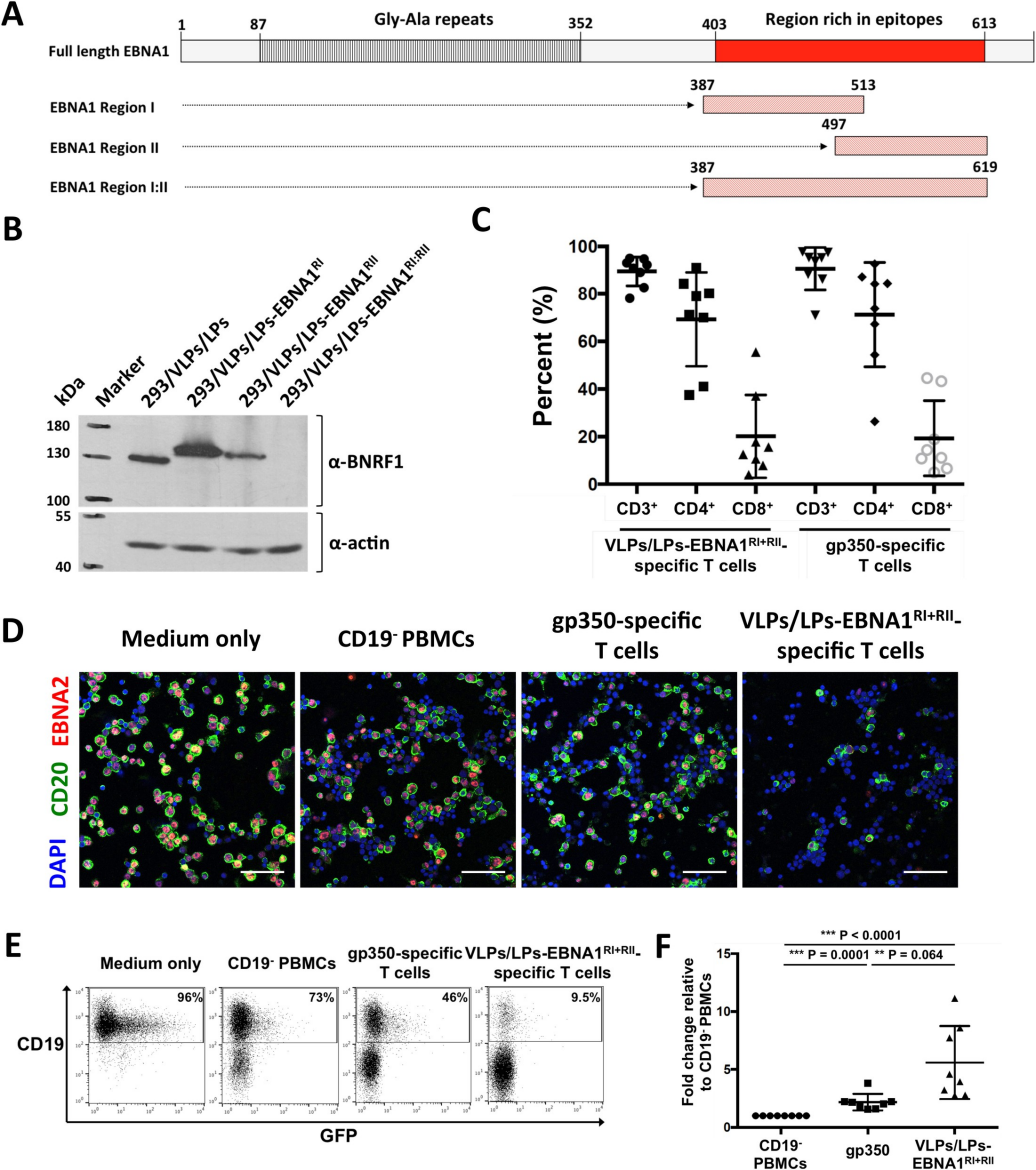
VLPs/LPs containing EBNA1 fragments expand T cells that efficiently target EBV-infected B cells. (A) Epitope-rich regions from EBNA1 were utilized to construct modified VLPs/LPs. EBNA1 has a single stretch of ~200 amino acids that contains more than 25 described CD4 and CD8 epitopes. Regions encompassing the majority of EBNA1 T-cell epitopes, arbitrarily named region I, II and I:II, were used to generate VLP/LPs-EBNA1^RI^, VLP/LPs-EBNA1^RII^ and VLP/LPs-EBNA1^RI:II^. The gly-ala repeats that impede HLA class I-restricted presentation is also shown. (B) Expression of BNRF1-EBNA1 fusion proteins by induced 293/VLPs/LPs-EBNA1^RI^, 293/VLPs/LPs-EBNA1^RII^ and 293/VLPs/LPs-EBNA1^RI:II^ producer cells. Western blot analysis was performed with α-BNRF1 and α-actin antibodies. (C) VLPs/LPs containing EBNA1 predominantly expand CD4^+^ T cells. VLPs/LPs-EBNA1^RI^ and VLPs/LPs-EBNA1^RII^ were combined in a 1:1 ratio (VLPs/LPs-EBNA1^RI+RII^) and used to stimulate PBMCs from eight unhaplotyped EBV-positive donors. The PBMCs from the same donors were stimulated in parallel with gp350-AgAb. *Ex vivo* cultures were stained for CD3, CD4 and CD8 after two stimulation cycles and analysed with flow cytometry. The percentage of CD4^+^, CD8^+^ and total T cells (CD3^+^) in *ex vivo* cultures are shown. (D-F) VLPs/LPs-EBNA1^RI+RII^-specific T cells efficiently control EBV-infected B cells during the first 5 days of infection. PBMCs from EBV-positive donors were stimulated as described in (C) and then cocultured with B cells that were infected overnight with recombinant B95-8. Additionally, infected B cells were cultured in medium only or with CD19-depleted (CD19^-^) PBMCs. *Ex vivo* cultures were analysed five days post-infection with immunofluorescence (D) and flow cytometry (E and F). (D) Representative immunofluorescence results from four donors are shown. Cells were stained with α-CD20, α-EBNA2 and DAPI prior to analysis. Scale bars represent 50 μm. (E) Representative flow cytometry data from eight donors are shown. *Ex vivo* cultures were assessed for the frequency of CD19^+^ cells. Percentages shown are of total cells. Since the recombinant B95-8 strain encodes GFP, CD19^+^GFP^+^ double-positive cells could be observed. (F) A summary of flow cytometry results from eight donors. The percentage of CD19^+^ cells in all cultures are expressed relative to the percentage of CD19^+^ cells in the presence of CD19^-^ PBMCs. Statistical analysis was performed using a two-tailed student t-test.

Since EBV-specific T cells play a crucial role in controlling EBV-infection [25,26], we tested whether modified VLPs/LPs could expand EBV-specific T cells with protective value. To this end, epitope-rich regions from EBNA1, arbitrarily named region I, region II and region I:II (Fig 3A), were used to generate VLPs/LPs producer cells that encode EBNA1 (S4 Fig). Analysis of the producer cells with western blot showed that the 293/VLP/LP-EBNA1^RI:II^ producer cell line was unable to express the large BNRF1-EBNA1 fusion, whilst the 293/VLP/LP-EBNA1^RI^ and 293/VLP/LP-EBNA1^RII^ producer cells successfully expressed their BNRF1-EBNA1 fusions (Fig 3B). Hence VLPs/LPs-EBNA1^RI:II^ were excluded from analysis. VLPs/LPs-EBNA1^RI^ and VLPs/LPs-EBNA1^RII^ were combined (VLPs/LPs-EBNA1^RI+RII^) and used to stimulate bulk PBMCs from unhaplotyped EBV-positive donors (Fig 3C). As a control, PBMCs from the same donors were also expanded with an antigen-armed antibody (AgAb) that contained the major EBV glycoprotein gp350. AgAbs were originally developed as a targeted therapy for B cell malignancies [18,27], but were repurposed in the present study to expand EBV-specific T cells of interest (S5 Fig). Stimulation of PBMCs from EBV-positive donors with VLPs/LPs-EBNA1^RI+RII^ or gp350-AgAb expanded similar numbers of CD4^+^, CD8^+^ and total T cells from the PBMCs of EBV-positive donors (Fig 3C). Next, primary B cells from four donors were infected with highly infectious gp110^high^ B95-8 [28] and then cocultured with VLPs/LPs-EBNA1^RI+RII^- or gp350-AgAb-expanded T cells. After 5 days, *ex vivo* cultures were analyzed by immunofluorescence for the presence of EBV-infected B cells (Fig 3D). This showed that nearly all the B cells in *ex vivo* cultures were EBNA2-positive, confirming that virtually all the B cells were successfully infected with EBV. However, there were noticeably fever EBV-infected B cells (CD20^+^EBNA2^+^) in the presence of VLPs/LPs-EBNA1^RI+RII^-specific T cells than gp350-specific T cells. This suggested that VLPs/LPs-EBNA1^RI+RII^-specific T cells were more efficient at controlling the EBV-infected B cells during the first five days of infection compared to gp350-specific T cells. Next, VLPs/LPs-EBNA1^RI+RII^- and gp350-AgAb-expanded T cells from eight donors were cocultured with infected B cells as before and quantitatively analysed with flow cytometry (Fig 3E and 3F). This confirmed that *ex vivo* cultures containing VLPs/LPs-EBNA1^RI+RII^-specific T cells had the lowest percentage of CD19^+^ cells. This indicates that VLPs/LPs-EBNA1^RI+RII^-specific T cells are more proficient than gp350-specific T cells at controlling EBV-infected B cells during the first five days of infection.

### Modified VLPs/LPs expand T cells that restrict the outgrowth of B95-8- and M81-infected B cells

**Fig 4:**
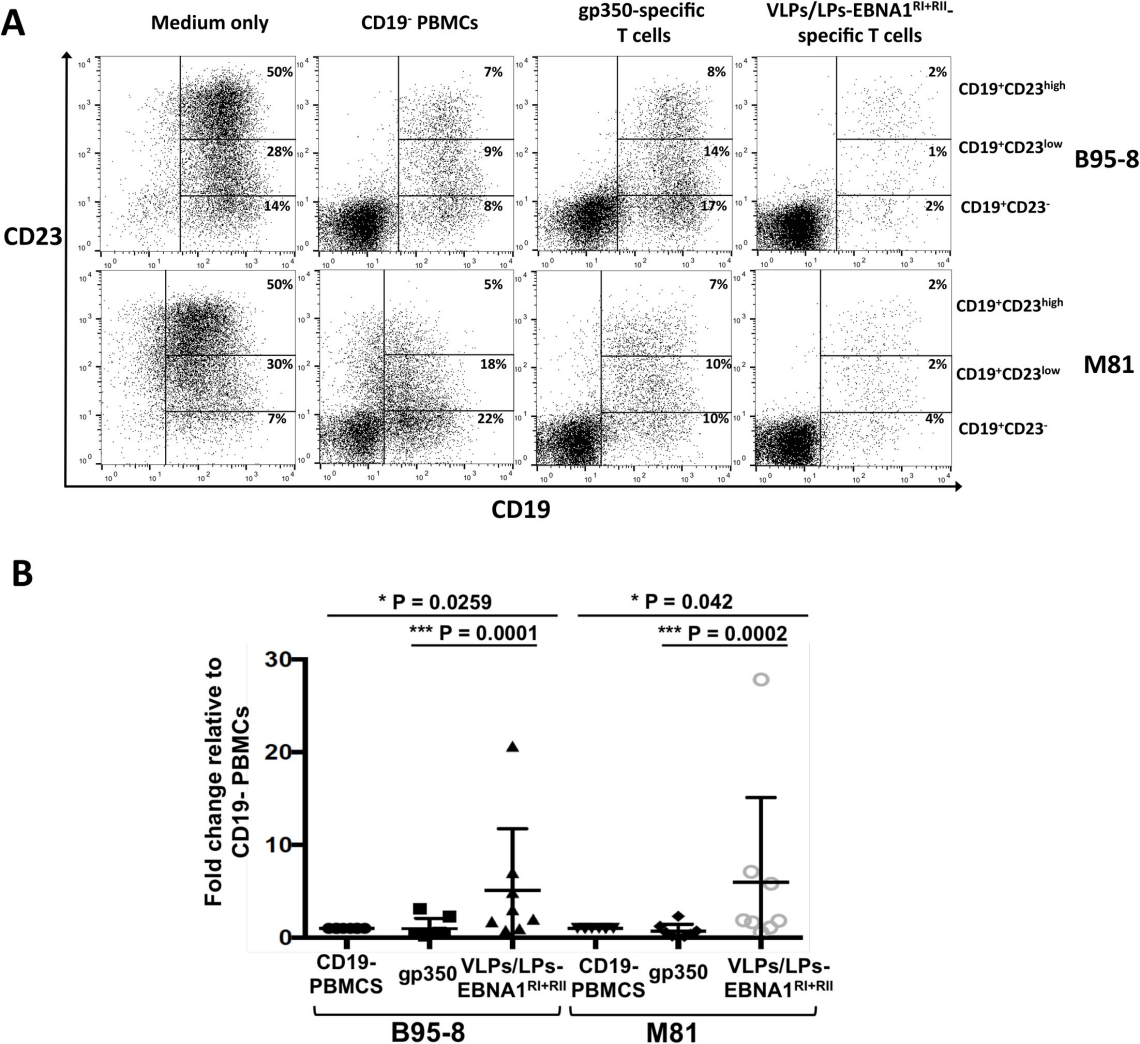
VLPs/LPs-EBNA1^RI+RII^-specific T cells prevent the outgrowth of B95-8- and M81-infected B cells. PBMCs from eight unhaplotyped EBV-positive donors were stimulated with gp350 or VLPs/LPs-EBNA1^RI+RII^ as described in Fig 3, and then cocultured with primary B cells that were infected with B95-8 or M81. In parallel, the infected B cells were cultured in medium only or with CD19^-^ PBMCs. After 15 days, *ex vivo* cultures were stained for CD19 and CD23 and then analysed by flow cytometry. (A) Representative flow cytometry data from eight donors. The percentage of CD19^+^CD23^-^, CD19^+^CD23^low^ and CD19^+^CD23^high^ B cells in *ex vivo* cultures are shown. (B) A summary of data obtained from eight donors. The percentage of CD19^+^CD23^+^ B cells in all cultures are expressed relative to the percentage of CD19^+^CD23^+^ B cells in the presence of CD19^-^ PBMCs. Statistical analysis was performed using a two-tailed student t-test. Only P values lower than 0.05 are shown.

Having shown that VLP/LPs-EBNA1^RI+RII^-specific T cells efficiently control B95-8-infected B cells, we tested whether they could restrict the outgrowth of infected B cells over a longer period. Additionally, we tested whether VLP/LPs-EBNA1^RI+RII^-specific T cells could prevent the outgrowth of B cells infected with the prototypic B95-8 strain or the distantly related M81 strain [29] from Hong Kong. We stimulated PBMCs from eight EBV-positive donors as before (see Fig 3) and then cocultured them with autologous B cells that were infected with gp110^high^ B95-8 and gp110^high^ M81. As a positive and negative control, infected B cells were respectively cultured with CD19^-^ PBMCs or in medium only. After 15 days, *ex vivo* cultures were analysed with flow cytometry to detect outgrowing B cells (Fig 4). Since proliferating B cells express CD23, outgrowing B cells were identified by detecting CD19^+^CD23^+^ double-positive cells [30,31]. EBV-infected B cells were found to consist of CD19^+^CD23^-^, CD19^+^CD23^low^ and CD19^+^CD23^high^ populations when they were cultured in medium only, with the majority of B cells being of the CD19^+^CD23^high^ variety (Fig 4A). Comparatively, in the presence of CD19^-^ PBMCs, gp350-specific T cells and VLPs/LPs-EBNA1^RI+RII^-specific T cells, the number of CD19^+^CD23^+^ cells were considerably reduced. This indicated that proliferating B cells were restricted in these cultures. Interestingly, whilst gp350-specific T cells were shown to be more efficient than CD19^-^ PBMCs at controlling infected B cells during the early phase of infection (see Fig 3), the CD19^-^ PBMCs of several donors were considerably more adept at restricting B-cell outgrowth than gp350-specific T cells over the longer 15 day period (Fig 4B). This suggest that the PBMCs from some donors contained EBV-specific T cells, other than gp350-specific T cells, that were able to restrict B-cell outgrowth. However, it is evident that proliferating B cells were restricted to a greater degree in *ex vivo* cultures that contained VLPs/LPs-EBNA1^RI+RII^-specific T cells. Moreover, this was observed for B95-8- and M81-infected B cells and for all donors (Fig 4B). This confirms that VLPs/LPs equipped with EBNA1 expand EBV-specific T cells that efficiently restrict B cells infected with B95-8 and M81 EBV.

### Modified VLPs/LPs stimulate cytolytic CD4^+^ T cells that recognize lytic and latent cycle antigens

**Fig 5:**
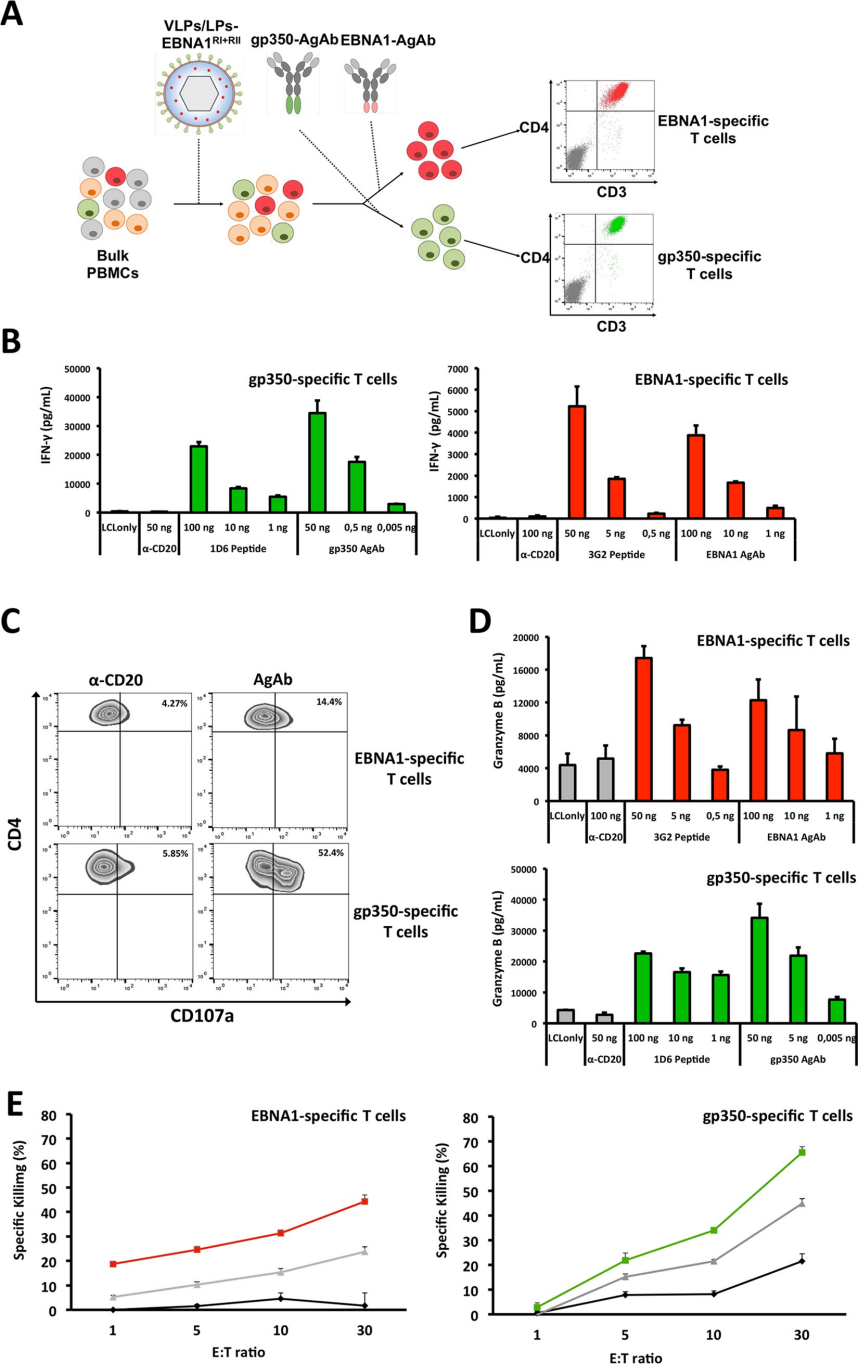
VLPs/LPs containing EBNA1 enable the expansion of cytolytic gp350- and EBNA1-specific CD4^+^ T cells. (A) The expansion of gp350- and EBNA1-specific T cells using VLPs/LPs-EBNA1^RI+RII^ and AgAb. PBMCs from EBV-positive individuals contain multiple EBV-specific T cells. This includes T cells that recognize EBNA1 (red cells), gp350 (green cells) or other structural antigens (orange cells). Stimulation of PBMCs with VLPs/LPs-EBNA1^RI+RII^ expanded these various T cells. Thereafter, EBNA1- and gp350-specific T cells were selectively expanded by respectively using EBNA1-AgAb and gp350-AgAb. *Ex vivo* expanded T cells were stained for CD3 and CD4 and analysed with flow cytometry. EBNA1- (red) and gp350- (green) specific T cells are shown. Unstained cells are shown in grey. (B) The *ex vivo* expanded CD4^+^ T cells are specific for EBNA1 or gp350. Autologous LCLs were pulsed with EBNA1-AgAb, gp350-AgAb, EBNA1 3G2 (514–528 aa) epitope or gp350 1D6 (65–69 aa) epitope and then cocultured with the CD4^+^ T cells. The release IFN-γ was quantified by ELISA. Each data point is the average of three values and error bars represents standard deviation. Each experiment is a representative of at least three. (C) EBNA1- and gp350-specific CD4^+^ T cells express CD107a. Autologous LCLs were pulsed with α-CD20, EBNA1-AgAb or gp350-AgAb and then cocultured with the CD4^+^ T cells. Thereafter, cells were stained for CD4 and CD107a and then analysed with flow cytometry. The percentage of CD4^+^ T cells that express CD107a are indicated. The data displayed are representative of three experiments. (D) EBNA1- and gp350-specific CD4^+^ T cells release granzyme B. Autologous LCLs were pulsed with EBNA1-AgAb, gp350-AgAb or relevant peptides (EBNA1 3G2 and gp350 1D6) and then cocultured with the CD4^+^ T cells. The release of granzyme B was quantified with an ELISA. The data displayed in each chart represent triplicate values and error bars represent standard deviation. Each graph is a representative experiment of at least three. (E) EBNA1- and gp350-specific CD4^+^ T cells lyse target cells pulsed with VLPs/LPs-EBNA1^RI+RII^. Autologous LCLs were pulsed overnight with VLPs/LPs- EBNA1^RI+RII^ (gray line), EBNA1 3G2 (red line), gp350 1D6 (green line) or EBNA3C 5H11 (black line) peptides. Thereafter, the pulsed LCLs were incubated with calcein AM and then cocultured with increasing amounts of the EBNA1- or gp350-specific CD4^+^ T cells. Effector to target (E:T) ratios of 1 to 30 were used. The release of calcein from targeted cells was measured at 535 nm after excitation with 485 nm light. Each data point is the average of three values and error bars represents error bars. Furthermore, each experiment is a representative of two experiments.

Having shown that VLPs/LPs-EBNA1^RI+RII^-specific T cells control EBV-infected cells, it indicated that they were cytolytic in character. However, since VLPs/LPs-EBNA1^RI+RII-^stimulated PBMCs contained primarily CD4^+^ T cells (Fig 3C), it was unclear whether VLPs/LPs-EBNA1^RI+RII^ are capable of stimulating EBV-specific CD8^+^ T cells. To this end, we tested the ability of LCLs pulsed with VLPs/LPs-EBNA1^RI^, containing the EBNA1 HPV CD8^+^ epitope, to stimulate an EBNA1 HPV-specific T-cell clone [18]. This showed that the EBNA1 HPV-specific CD8^+^ T-cell clone was unable to recognize LCLs pulsed with VLPs/LPs-EBNA1^RI^ (S6A Fig). In contrast, LCLs pulsed with the VLPs/LPs-EBNA1^RI^ were well recognized by the BNRF1 VSD-specific CD4^+^ T-cell clone. This suggests that the autologous LCLs were unable to cross-present the EBNA1 HPV epitope from VLPs/LPs-EBNA1^RI^ to CD8^+^ T cells. We also assessed IFN-gamma secretion by CD4^+^ and CD8^+^ T cells after the stimulation of bulk PBMCs with VLPs/LPs-EBNA1^RI+RII^ (S6B Fig). This revealed that both CD4^+^ and CD8^+^ IFN-gamma producing cells were detected upon stimulation with VLPs/LPs-EBNA1^RI+RII^ (S6C Fig). However, it was evident that IFN-γ^**+**^ CD4^+^ T cells were more numerous than IFN-γ^**+**^ CD8^+^ T cells and suggests that the stimulation of PBMCs with VLPs/LPs-EBNA1^RI+RII^ preferentially expands EBV-specific CD4^+^ T cells. Next, we expanded EBNA1- and gp350-specific CD4^+^ T cells from VLPs/LPs-EBNA1^RI+RII^-stimulated PBMCs and analyzed them for their cytotoxic potential. Bulk PBMCs from an unhaplotyped EBV-positive donor was stimulated with VLPs/LPs-EBNA1^RI+RII^ for two rounds, after which gp350-AgAb (S5 Fig) or EBNA1-AgAb (S7 Fig) were used to expand gp350- and EBNA1-specific CD4^+^ T cells (Fig 5A). The expanded CD4^+^ T cells were confirmed to be specific for either EBNA1 or gp350 (Fig 5B). The *ex vivo* expanded CD4^+^ T cells specifically responded to EBNA1-AgAb or gp350-AgAb and to EBNA1 3G2 or gp350 1D6 epitope peptides. Next, we determined whether the EBNA1- and gp350-specific CD4^+^ T cells were capable of expressing CD107a, a surrogate marker for the release of cytolytic granules [32]. Autologous LCLs were pulsed with α-CD20, EBNA1-AgAb or gp350-AgAb then cocultured with the EBNA1- and gp350-specific CD4^+^ T cells. This showed that both CD4^+^ T-cell lines upregulated CD107a in response to the relevant antigen (Fig 5C). However, approximately 50% of gp350-specific CD4^+^ T cells expressed CD107a, whilst only 10% of EBNA1-specific CD4^+^ T cells expressed CD107a. Next, we tested the ability of EBNA1- and gp350-specific CD4^+^ T cells to release the mediator of cytolysis granzyme B (Fig 5D). Both the EBNA1- and gp350-specific CD4^+^ T cells released granzyme B in response to the relevant AgAb and epitope peptide. Lastly, we tested whether the EBNA1- and gp350-specific CD4^+^ T cells were capable of directly lysing autologous LCLs pulsed with antigen (Fig 5E). This showed that the both the EBNA1- and gp350-specific CD4^+^ T cells specifically lysed LCLs pulsed with epitope peptides and VLPs/LPs that contained EBNA1. Taken together, these results confirm that VLPs/LPs-EBNA1^RI+RII^ have the ability to stimulate cytolytic CD4^+^ T cells specific for lytic and latent antigens. These results are consistent with previous studies that showed EBNA1- [30,33,34] and gp350- [35] specific T cells to be cytolytic.

### Vaccination with EBV VLPs/LPs containing EBNA1 protects mice from wtEBV infection

**Fig 6:**
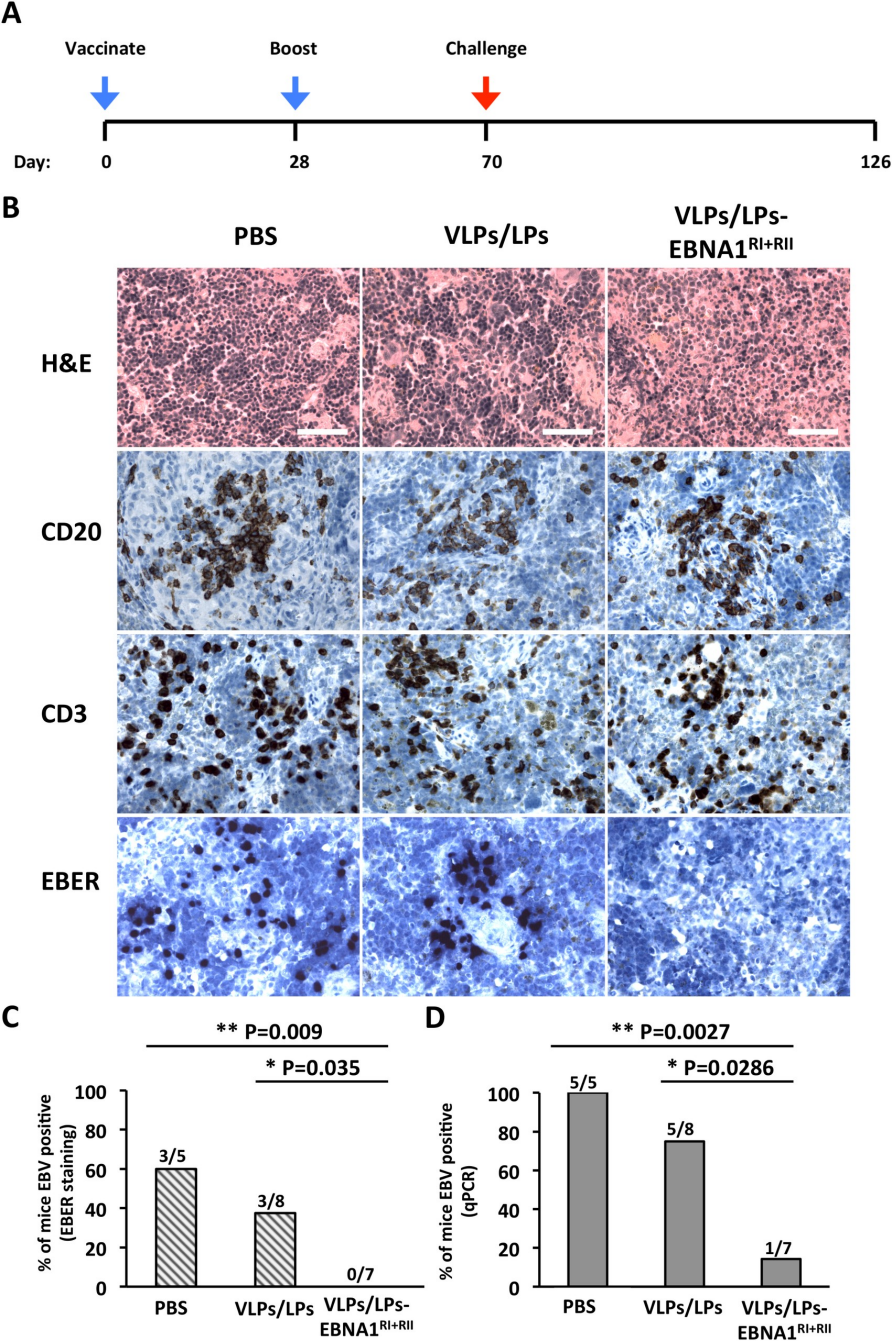
Vaccination of humanized mice with VLPs/LPs-EBNA1^RI+RII^ confers protective immunity. (A) Immunization schedule of humanized NSG-A2 mice and challenge with wtEBV. Three groups of mice were vaccinated and boosted with PBS (n = 5), VLPs/LPs (n = 8) or VLPs/LPs-EBNA1^RI+RII^ (n = 7), with poly (I:C) serving as adjuvant. Mice were challenged with B95-8 (GRU = 1 x10^5^) six weeks after the boost. After eight weeks, all animals were euthanized and their tissues analysed for evidence of EBV infection. (B) Histological analysis of mice spleens after challenge with wtEBV. Spleen sections of mice were stained with H&E, α-human CD20 antibody, α-human CD3 antibody and a probe specific for EBER non-coding RNAs. Scale bars represent 50 μm. (C) Incidence of EBV infection based on EBER staining of spleens. (D) Incidence of EBV-infection based on real-time qPCR analysis of peripheral blood. Statistical analysis was performed on the results shown in (C) and (D) using a one-tailed Chi-square test. Only P values lower than 0.05 are shown.

Having shown that modified VLPs/LPs were antigenic *in vitro* and *ex vivo*, we assessed whether VLPs/LPs- EBNA1^RI+RII^ had protective abilities *in vivo*. To this end, mice reconstituted with human immune system components, susceptible to EBV infection and capable of exerting EBV-specific immune control [36], were used to interrogate VLPs/LPs- EBNA1^RI+RII^. Humanized NSG-A2 (huNSG-A2) mice were randomly grouped and injected intraperitoneally with PBS, unmodified VLPs/LPs (1 x 10^6^ particles) or VLPs/LPs-EBNA1^RI+RII^ (1 x10^6^ particles), using poly (I:C) as an adjuvant (Fig 6A). Four weeks later, mice were boosted using the same dose. Animals were challenged with gp110^high^ B95-8 (1 x 10^5^ GRUs) six weeks after the last boost and euthanized eight weeks later. From the literature we knew that this titer would enable infection without gross development of tumors [36]. The spleens of challenged animals were analysed by histology (Fig 6B). This showed that all animals contained human CD20- and CD3-positive cells in their spleens. However, there was no correlation between the abundance of CD20- and CD3-positive cells and the different treatments. *In situ* hybridization revealed the presence of interspersed cells that expressed EBV-encoded RNAs (EBERs) in the spleens of mice from the PBS and unmodified VLPs/LPs groups. In total, 60% of mice from the PBS group were found to contain EBER^+^ cells, while 37.5% of the mice from the VLPs/LPs group contained EBER^+^ cells (Fig 6C). Statistical analysis showed that this observation was statistically insignificant (P > 0.05). None of the spleen samples from the VLPs/LPs-EBNA1^RI+RII^ group were found to contain EBER^+^ cells. This result was confirmed to be statistically significant from the PBS (P = 0,009) and VLPs/LPs (P = 0.035) groups. Next, qPCR was used to detect the presence of EBV in the peripheral blood of challenged animals (Fig 6D). This showed that 100% of mice from the PBS group contained EBV DNA in their peripheral blood, compared to 62% of the VLPs/LPs group and 14% of the VLPs/LPs-EBNA1^RI+RII^ group. Once more, statistical analysis revealed that the observed difference between the PBS and VLPs/LPs group was not significant (P > 0.05). However, statistical analysis showed that the difference between the VLPs/LPs-EBNA1^RI+RII^ group and the PBS (P = 0.0017) and VLPs/LPs (P = 0.0286) groups was significant. In summary, this indicates that vaccination with VLPs/LPs-EBNA1^RI+RII^ afforded significantly better protection during the eight-week challenge period than vaccination with unmodified VLPs/LPs. However, whether vaccination with VLPs/LPs-EBNA1^RI+RII^ would offer long-term protection against EBV infection in humans remains unknown. Nonetheless, our results bode well for the development of second generation VLPs/LPs that contain multiple latent antigens.

## Discussion

DNA-free EBV VLPs/LPs are structurally complex, composed of multiple lytic gene products, incapable of infection and highly immunogenic [14]. Considering the breadth of EBV-specific immune responses in healthy individuals [10], it is likely that prophylactic vaccination would benefit from an immunogen that contains EBV-antigens that are expressed during lytic replication and latency. To this end, we used EBV VLPs/LPs as a platform to generate immunogenic particles that comprise lytic and latent antigens. Considering the importance of T-cell responses in controlling of EBV infection [37], we extensively assessed the ability of modified VLPs/LPs to stimulate EBV-specific T cells.

Modification of EBV VLPs/LPs to contain latent antigens successfully enlarged their antigenic spectrum, enabling the stimulation of several lytic protein- and latent protein-specific CD4^+^ T-cell clones. The antigenicity of EBV VLPs/LPs containing EBNA1 were also analyzed in an *ex vivo* setting by stimulating bulk PBMCs from EBV-positive donors to yield EBV-specific T-cell lines. *Ex vivo* generated T-cell lines contained EBV-specific CD4^+^ and CD8^+^ T cells, but it was evident that CD4^+^ T cells were more numerous. The preferential expansion of CD4^+^ T cells from bulk PBMCs is supported by earlier studies which have shown that EBV structural proteins are weakly immunogenic towards CD8^+^ T cells [38,39] and are instead immunodominant targets of CD4^+^ T cells [26]. The ability of B cells to efficiently present several structural proteins from incoming virions to CD4^+^ T cells [35,40] suggests that structural protein-specific CD4^+^ T cells are likely to play a more dominant role than CD8^+^ T cells during the very early stages of infection. However, the recent identification of BcLF1-specific T-cell clones that can recognize LCLs pulsed with UV-inactivated EBV [41] suggests that structural protein-specific CD8^+^ T cells may also play an important role during the early stage of infection. In contrast, we found that LCLs pulsed with modified EBV VLPs were unable to stimulate an EBNA1-specific CD8^+^ T-cell clone, implying that cross-presentation might be limited to particular structural antigens. Future studies will certainly shed light on this newly discovered phenomenon.

We have shown that T cells expanded with modified VLPs are considerably more efficient at controlling recently infected B cells compared to those expanded with gp350. Since VLPs/LPs contain multiple EBV antigens, they likely expanded a polyclonal pool of EBV-specific T cells that simultaneously recognize different antigenic epitopes displayed by infected B cells. Indeed, recently infected B cells have been shown to display multiple structural proteins [35,40]. The inability of gp350-specific T cells to competently target recently infected B cells has profound implications for prophylactic vaccine design, since T-cell responses would be of paramount importance if a small number of EBV virions are not neutralized and manage to infect a subset of cells. The major envelope glycoprotein gp350, arguably the most intensively studied EBV antigen, has been investigated as a prophylactic vaccine for several decades and continues to garner attention [6,11,12,42,43]. The continued focus on gp350 is sensible considering gp350 is chiefly responsible for attachment to B cells during the infection process and is a frequent target of neutralizing antibodies [44]. Indeed, emerging gp350 vaccines have improved in their ability to generate neutralizing antibodies [12,43]. However, the ability of gp350-specific T cells to provide optimal protection against recently EBV-infected cells has previously not been seriously questioned. Our analyses suggest that gp350-specific T cells alone are suboptimal for targeting EBV-infected B cells during the early phase of infection. This implies that prophylactic vaccines are more likely to generate protective T-cell responses during the early phase of EBV-infection if they include multiple lytic antigens. Additionally, prophylactic vaccines composed exclusively of gp350 would not elicit antibody responses that protect against epithelial cell infection. EBV infects epithelial cells independently of gp350 [45], a process recently shown to rely on gH/gL [46,47]. From this perspective, a vaccine composed of gH/gL has an advantage over gp350 since it may prevent epithelial and B cell infection [48]. Since VLPs/LPs contain both gp350 and gH/gL, along with several other EBV glycoproteins, they are likely to generate antibody responses that protects against B-cell and epithelial cell infection. Whilst we did not focus on antibody responses in the present study, the structural similarity of VLPs/LPs to wtEBV suggests they would enable the generation of neutralizing antibodies to several EBV glycoproteins in their natural context. Indeed, UV-irradiated EBV has previously been shown to generate potent neutralizing antibodies [43]. From this perspective, prophylactic vaccines that contain numerous EBV antigens, including glycoproteins that mediate B-cell and epithelial cell infection, are likely to stimulate superior protective antibody and T-cell responses.

Humoral and cellular immune responses that recognize multiple structural proteins are likely to provide a protective benefit by targeting virions, recently infected cells and cells undergoing reactivation [35,49,50]. However, they are unlikely to offer protect against latently infected cells. The establishment of type I, II or III latency in just a handful of cells would enable the number of EBV-infected cells to increase through simple cell division [51]. The inability of vaccination to protect against latently infected cells is especially important to consider since it is latency transcription programs that predominate during IM, EBV-associated lymphoproliferative disease and EBV-positive malignancies [52]. The ability of EBV-specific T cells to control EBV-infected cells and prevent post-transplant lymphoproliferative disease (PTLD) strongly suggests that prophylactic vaccination should elicit latent protein-specific T cells in addition to lytic protein-specific T cells [53–55]. Since the mouse challenge model in the present study extended for eight weeks, the antigenic load of structural proteins are likely to have been markedly reduced relative to that of latent proteins. Vaccination of humanized mice with VLPs containing EBNA1 afforded more protection against EBV infection than vaccination with unmodified VLPs/LPs. Since humanized mice produce little or no EBV-specific antibodies [36,56], it likely that protection against EBV infection did not involve humoral immunity. Rather, it is more probable that vaccination with VLPs/LPs-EBNA1^RI+RII^ elicited protective T cell responses. The recognition of structural proteins and EBNA1 possibly enabled the humanized mice to target EBV-infected cells during the whole EBV life cycle (except latency 0). Indeed, EBNA1 is the only latent protein that is expressed during all forms of EBV latency (except type 0) and in all EBV-positive malignancies [30]. Whether vaccination with lytic and latent EBV antigens can afford protection against EBV in humans remains to be determined.

Prototype EBV vaccine candidates have thus far not directly addressed the daunting task of affording protection against the plethora of EBV strains that exist worldwide. In recent years, the advent of high throughput sequencing has enabled the detailed analysis of various EBV strains [57]. This has revealed that EBV-encoded proteins can be highly polymorphic. Recently, T-cell epitopes from several EBV strains have been compared [58]. This revealed that epitopes differ considerably between different EBV strains, with almost 50% of CD4^+^ epitopes and almost 30% of CD8^+^ epitopes, including their flanking region, varying between the B95-8 and M81 strains. Whilst EBV strain heterogeneity presents a hurdle for T-cell immunity, our results show that polyclonal memory T cells stimulated with modified B95-8 VLPs/LPs can restrict B cells transformed with B95-8 and M81 at a relatively early stage of infection. However, it is unknown whether modified B95-8 VLPs/LPs would afford protection against M81 *in vivo*. Since latent genes are more polymorphic than lytic genes, it is possible that B95-8 latent antigens would not afford optimal protection against those encoded by other EBV strains. The identification of T-cell epitopes that are conserved amongst different EBV strains would certainly aid vaccine design efforts, enabling this hurdle to be overcome. Nonetheless, a vaccine consisting of multiple EBV proteins, possibly from different strains, is also likely to combat the problem of EBV strain heterogeneity.

EBV VLPs/LPs are likely to be further improved as a prophylactic vaccine through the inclusion of antigens in addition to EBNA1. Several interesting candidates, latent and lytic proteins, have recently been highlighted [40]. Modification of multiple tegument proteins will enable the incorporation of several antigens into EBV VLP/LPs. By incorporating the most immunodominant EBV antigens into VLPs/LPs it will enable them to prime the immune system against viral antigens that are expressed at all stages of infection and in all types of EBV-associated tumors, whilst enabling the generation of neutralizing antibodies against surface viral proteins. Such a multipronged approach is likely to increase the protection afforded by the EBV VLPs/LPs.

## Materials and methods

### Ethics statement

Peripheral blood mononuclear cells (PBMCs) were isolated from healthy donors that provided written informed consent (ethical approval granted by the Ethikkommission of the Medizinische Fakultät Heidelberg (S-603/2015)) or from anonymous buffy coats purchased from the *Institut für Klinische Transfusionsmedizin und Zelltherapie* (IKTZ) in Heidelberg and did not require ethical approval. Animal experiments were approved (approval number G156-12) by the federal veterinary office at the Regierungspräsidium Karlsruhe (Germany) and were performed in strict accordance with German animal protection law (TierSchG). Mice were handled in accordance with good animal practice, as defined by the Federation of European Laboratory Animal Science Associations (FELASA) and the Society for Laboratory Animal Science (GV-SOLAS), and were housed in the class II containment laboratory of the German Cancer Research Center.

### Cell lines and primary cells

Cell lines include EBV-positive Raji cells (ATCC CCL-86) [59], EBV-negative Elijah B cells (kindly provided by Prof. A.B. Rickinson), 293 cells (ATCC CRL-1573) [60], T cells specific for EBNA1 3E10, EBNA3C 5H11, gp350 1D6 and BNRF1 VSD epitopes (kindly provided by Prof. J. Mautner) and autologous LCLs (kindly provided by Prof. J. Mautner). Peripheral blood mononuclear cells (PBMCs) were isolated using Ficoll-Paque Plus and primary B cells were isolated using Dynabeads CD19 Pan B (Invitrogen) and DETACHaBEAD CD19 kit (Invitrogen). RPMI containing 10% fetal calf serum (F7524, Sigma) was used to culture 293, Raji and Elijah cells. T-cell clones and lines were cultured as previously described [17,35].

### Construction and production of AgAbs

AgAbs were constructed using sequences coding for EBNA1 (390–622 aa) and gp350 (1–470 aa). Latent protein-coding sequences were PCR amplified and introduced downstream of an α-CD20 HC gene contained within the pRK5 expression vector [18]. The primers used to construct AgAbs are listed in S1 Table. The α-CD20 antibody and AgAbs were produced by transfecting the appropriate heavy chains and the α-CD20 light chain into 293 cells using polyethylenimine (PEI). The following day the PEI-containing medium was removed and replaced with serum-free FreeStyle 293 expression medium and cells were incubated for three days. Supernatants were centrifuged at 400 x g for 10 minutes and filtered through a 0,22 μm filter.

### Recombinant BAC DNA and stable producer cell lines

Recombinant BAC DNA was constructed using galK recombination [61]. In the present study, wtEBV (B95-8) [62] or VLPs/LPs (B95-8ΔBFLF1/BFRF1a/BALF4) [21] BAC DNA were modified to encode latent protein fragments. Only VLPs/LPs lacking BALF4, encoding the glycoprotein gp110, were utilized in the present study due to their enhanced safety [20,21]. The primers used for the construction of BAC mutants, as well as a description of all BAC mutants, are shown in S1 Table. The first step in galK recombination was the insertion of the galK cassette into the BNRF1 ORF of wtEBV or VLPs/LPs BAC DNA. Subsequently, the galK cassette was replaced with DNA fragments encoding latent protein moieties. Outgrowing colonies were analysed with restriction digestion and sequencing to confirm the integrity of BNRF1-latent protein fusions. Stable producer cells were generated with the recombinant BAC DNA as previously described [63].

### Production of virus and VLPs/LPs

An expression plasmid encoding BZLF1 (p509) was transfected into producer cells to induce virus or VLPs/LPs production [14]. For the production of EBV (B95-8 or M81) for *ex vivo* and *in vitro* studies, the pRA plasmid encoding gp110 was cotransfected with p509 for increased infectivity [28]. The liposome-based transfectant Metafectene (Biontex) was used to carry out transfections overnight. Subsequently, Metafectene-containing medium was removed and replaced with fresh medium. Transfected cells were incubated for three days before supernatants were harvested. Supernatants were centrifuged at 400 x g for 10 minutes and filtered through a 0.44 μm filter. VLPs/LPs used for *ex vivo* T-cell expansions and animal experiments were produced in serum-free FreeStyle 293 expression medium (Gibco). In all other cases, virus and VLPs/LPs were produced on RPMI supplemented with 10% FCS. Lastly, virus and VLPs/LPs used in animal experiments were concentrated at 18 000 x g for 3 hours and resuspended in PBS.

### Real-time qPCR

Virus titers were determined by real-time qPCR as previously described [14]. In brief, virus-containing supernatants were treated with DNase I (5 units) and proteinase K (1mg/mL). Next, real-time qPCR analysis was carried out using primers and probe specific for the EBV BALF5 gene. To determine the presence of EBV in peripheral blood, genomic DNA from vaccinated and challenged animals was compared to unchallenged animals.

### Quantification of VLPs/LPs with flow cytometry

wtEBV, previously quantified with real-time qPCR, was titrated (1, 0.75, 0.5, and 0.25 x 10^7^ geq) and bound to Elijah B cells at 4°C. Cells were washed, stained with α-gp350 (clone 72A1) and α-mouse IgG-Cy3 antibodies and analysed with flow cytometry. MFI values were determined for different amounts of virus. A standard curve was generated for EBV genomes vs MFI. Concurrently, supernatants containing VLPs/LPs were incubated with Elijah B cells and stained as above. MFI values obtained for VLPs/LPs were extrapolated off the standard curve to quantify VLPs/LPs.

### T-cell activation assays

IFN-γ in cell culture supernatants was determined as previously described [18]. Autologous LCLs were pulsed overnight with antigen and cocultured for a minimum of 18 hours with T-cells at an E:T ratio of 1:1. Supernatants were analysed by ELISA (Mabtech). In blocking studies with neutralizing antibody (72A1 clone), virus containing supernatants were preincubated with antibody for 1 hour at 37°C before being used in T-cell activation assays.

### Short-term *ex vivo* stimulation of PBMCs with VLPs/LPs-EBNA1^RI+RII^ or gp350-AgAb

Bulk PBMCs from EBV-positive donors were pulsed with VLPs/LPs-EBNA1 (1 x 10^6^ particles) or gp350-AgAb (20 ng). After two days, cultures were supplemented with IL-2 (10 U/mL) and thereafter maintained in medium containing IL-2. Cells were restimulated 10 days later using IL-2 (10 U/mL) and the same amount of VLPs/LPs-EBNA1 or gp350-AgAb. One week later, cells were analysed for the presence of CD4, CD8 and CD3 expressing cells or where cocultured with primary B cells that were infected overnight with EBV.

### Targeting of recently infected B cells by VLPs/LPs-EBNA1-stimulated PBMCs

Bulk PBMCs from four EBV-positive donors were stimulated for two rounds with VLPs/LPs-EBNA1 (1 x 10^6^ particles) or gp350-AgAb (20 ng) in the presence of IL-2 (10 U/mL). Autologous primary B cells were infected overnight with B95.8 (MOI = 3) and then cocultured with the stimulated PBMCs, CD19^-^ depleted PBMCs or medium only. *Ex vivo* cultures were analysed with flow cytometry and immunofluorescence 5 days post-infection to observe EBV-positive cells. Cells were stained with α-CD19-APC (HIB19 clone) prior to flow cytometry and α-CD20 (L26 clone), α-EBNA2 (PE2 clone) and DAPI prior to immunofluorescence.

### Restriction of B cell outgrowth by VLPs/LPs-EBNA1-stimulated PBMCs

Bulk PBMCs from eight EBV-positive donors were stimulated for two rounds with VLPs/LPs-EBNA1 (1 x 10^6^ particles) or gp350-AgAb (20 ng) in the presence of IL-2 (10 U/mL). B cells were infected with B95-8 or M81, respectively using an MOI of 3 or 30 to account for their different transforming abilities [29]. *Ex vivo* cultures stained with α-CD19-APC (HIB19 clone) and α-CD23-PE-Cy7 (EBVCS2 clone) antibodies and analysed by flow cytometry.

### Expansion of EBNA1 and gp350-specific CD4^+^ T cells from VLPs/LPs-EBNA1-stimulated PBMCs

PBMCs from an EBV-positive donor were stimulated for one round with VLPs/LPs-EBNA1^RI+RII^ (1 x 10^6^ particles) in the absence of IL-2. After two weeks, cells were restimulated using irradiated (40 Gy) autologous PBMCs, pulsed with the same dose of VLPs/LPs-EBNA1^RI+RII^, in the presence of IL-2. After another two weeks, EBNA1- or gp350-specific T cells were expanded by stimulating cells biweekly with AgAbs (10–50 ng) that contained EBNA1 or gp350. Autologous LCLs, generated using B95-8ΔZR, were used as antigen presenting cells after the fifth round of stimulation. T cells were maintained in AIM V medium supplemented with 10% pooled human serum, IL-2 (10 U/mL), 10 mM HEPES, 2 mM L-glutamine, 50 μg/mL gentamicin and 0.4 mg/ mL ciprofloxacin.

### Intracellular IFN-gamma staining

Bulk PBMCs (5 x 10^6^ cells) from EBV-positive donors were pulsed with VLPs/LPs-EBNA1^RI+RII^ (1 x 10^6^ particles) and supplemented with IL-2 (10U/mL) two days later. After another six days, cultures were restimulated with medium, EBNA1 peptide (PepTivator, Miltenyi), gp350-AgAb (20 ng) or VLPs/LPs-EBNA1^RI+RII^ (1 x 10^6^ particles) in the presence of bredfeldin A (Biolegend) for 4,5 hours. Cells were stained with α-CD4-APC (RPA-T4 clone) and α-CD8-PE-Cy7 (RPA-T8), fixed/permeabilized (Fixation/Permeabilization solution kit, BD Biosciences), stained with α-IFN-γ-PE (B27 clone) and analysed by flow cytometry.

### Generation, vaccination and challenge of humanized NSG-A2 mice

NSG-A2 mice (NOD.Cg-Prkdc^scid^Il2rg^tm1Wjl^Tg (HLA-A2.1) 1Enge/SzJ) were humanized with CD34^+^ hematopoietic progenitor cells (HPCs) that were isolated from human fetal liver tissue (Advanced Bioscience Resources, USA) [64]. Newborn mice were irradiated (1 Gy) and injected intrahepatically with CD34^+^ HPCs. After twelve weeks, the presence of human CD45^+^ cells in the peripheral blood of mice was determined to confirm successful humanization. In total, 20 humanized NSG-A2 (huNSG-A2) mice were randomly grouped according to similarity of humanization ratios and injected intraperitoneally in a single blind fashion with PBS, VLPs/LPs (1 x 10^6^ particles) or VLPs/LPs-EBNA1^RI+RII^ (1 x 10^6^ particles). In all cases, 50 μg poly (I:C) was used as adjuvant. Animals were boosted one month later with the same treatments. One and a half months after the boost, animals were injected intraperitoneally with 1 x 10^5^ GRUs of B95-8. Mice were sacrificed eight weeks post-infection and their blood and tissues analysed for evidence of EBV infection [64]. All the VLPs/LPs and virus used in animal experiments were obtained by centrifuging supernatants at 18 000 x g for 3 hours and resuspending in PBS.

## Supporting information

S1 Fig Construction of EBV BAC DNA encoding a BNRF1-latent protein fusion. (A) Galk recombination was carried out with a 150 bp fragment corresponding to 320–344 aa and 633–656 aa of EBNA3C. *Eco*RI and *Bam*HI restriction sites before and after recombination are shown, as are the size of fragments generated by these enzymes. (B) Restriction digestion with *Eco*RI and *Bam*HI confirmed that EBV-E3C BAC DNA from 293 producer cells generated the same restriction fragments as EBV-E3C BAC DNA constructed in *E*.*coli*. White arrows emphasize DNA fragments that are different between wtEBV DNA (B95-8) and DNA modified with galK recombination.
(TIF)

S2 Fig Constructing EBV and VLP/LP BAC DNA encoding a BNRF1-EBNA3C-EBNA1 fusion protein. A 300 bp fragment encoding the EBNA3C (E3C) (320–344 aa and 633–656 aa) and EBNA1 (E1) (476–505 aa and 522–546 aa) was introduced into EBV (A) and VLP/LP (B) BAC DNA using galK recombination. *Eco*RI and *Bam*HI restriction sites before and after recombination are shown, as well as the size of fragments generated by these enzymes. Restriction digestion with *Eco*RI and *Bam*HI confirmed that EBV-E3C-E1 (C) and VLP/LP-E3C-E1 (D) BAC DNA from 293 producer cells generated the same restriction fragments as BAC DNA constructed in *E*.*coli*. White arrows emphasize DNA fragments that are different between unmodified EBV and VLP/LP DNA modified with galK recombination.
(TIF)

S3 Fig Quantification of VLPs/LPs using flow cytometry. (A) The ability of VLPs/LPs and wtEBV to bind B cells was exploited for quantification purposes. B cells exposed to wtEBV or VLPs/LPs were stained with α-gp350 (72A1) and α-mouse IgG-Cy3 antibodies and analysed with flow cytometry. (B) wtEBV, previously quantified with qPCR, was added in increasing amounts (geq) to B cells and analysed with flow cytometry. This revealed a linear relationship between virus titer (geq) and median fluorescence intensity (MFI). Similarly, the MFI was determined for VLPs/LPs-E3C-E1 and the linear relationship between MFI and virus titer used for their quantification.
(TIF)

S4 Fig Construction of VLP/LP BAC DNA encoding BNRF1-EBNA1 fusion proteins. VLP/LP BAC DNA was modified with galK recombination using EBNA1-coding sequences corresponding to region I (A), II (B), and I:II (C). *Eco*RI restriction sites and restriction fragments are shown. Restriction digestion of BAC DNA with *Eco*RI confirmed that VLP/LP-EBNA1^RI^ (D), VLP/LP-EBNA1^RII^ (E) and VLP/LP-EBNA1^RI:II^ (F) from producer cells was the same as BAC DNA constructed in *E*.*coli*. White arrows highlight DNA fragments that are different between unmodified VLP/LP BAC DNA and galK modified VLP/LP BAC DNA.
(TIF)

S5 Fig Validation of gp350-AgAb as a tool for expanding gp350-specific T cells *ex vivo*. The ligand-binding domain (1–470 aa) of gp350 was fused to the CH3 domain of α-CD20, expressed in 293 cells and used to stimulate (dotted lines) the PBMCs from an unhaplotyped EBV-positive donor in the presence of IL-2. After a minimum of six stimulation cycles, *ex vivo* cultures were stained for CD3 and CD4 and analysed by flow cytometry. The percentage of CD3^+^CD4^+^ double-positive cells in *ex vivo* cultures is shown. Unstained cells are shown in grey. A T-cell activation assay was performed to confirm that the expanded T cells were specific for gp350-AgAb. Autologous LCLs were pulsed with medium, unmodified α-CD20 or gp350-AgAb and then cocultured with the CD4^+^ T cells. The release of IFN-γ was measured by ELISA.
(TIF)

S6 Fig Modified VLPs/LPs predominately stimulate CD4^+^ T cells. (A) Autologous LCLs were pulsed with unmodified VLPs/LPs (1 x 10^6^ particles) or VLPs/LPs-EBNA1^RI^ (1 x 10^6^ particles) and then cocultured with T cells specific for the CD4-restricted BNRF1 VSD epitope (1006–1017 aa) or the CD8-restricted EBNA1 HPV epitope (407–417 aa). T-cell activity was determined by quantifying IFN-γ release with ELISA. The assay was performed in triplicate and standard deviations are illustrated. (B) PBMCs from EBV-positive donors were stimulated with VLPs/LPs-EBNA1^RI+RII^ for a single round and the frequencies of IFN-γ^+^CD8^+^ (top row) and IFN-γ^+^CD4^+^ (bottom row) T cells were determined after restimulation with medium, EBNA1 peptide, gp350-AgAb and VLPs/LPs-EBNA1^RI+RII^. Representative data from six experiments are shown and displayed percentages are of total cells. (C) A summary of IFN-γ secretion from six donors. Statistical analysis was performed using a two-tailed student t-test. Only P values lower than 0.05 are shown.
(TIF)

S7 Fig Validation of EBNA1-AgAb as a tool for expanding EBNA1-specific T cells *ex vivo*. An epitope-rich region of EBNA1 (390–622 aa) was fused to the CH3 domain of α-CD20, expressed in 293 cells and used to stimulate (dotted lines) the PBMCs from an unhaplotyped EBV-positive donor in the presence of IL-2. After a minimum of six stimulation cycles, *ex vivo* cultures were stained for CD3 and CD4 and analysed with flow cytometry. The percentage of CD3^+^CD4^+^ double-positive cells are shown. Unstained cells are shown in grey. A T-cell activation assay was performed to confirm the specificity of the expanded T cells towards the EBNA1-AgAb. Autologous LCLs were pulsed with medium, unmodified α-CD20 or EBNA1-AgAb and then cocultured with the CD4^+^ T cells. The release of IFN-γ was measured by ELISA.
(TIF)

S1 Table List of oligonucleotides. (PDF)
