# Supplementary figures and images for: Immunogenic particles with a broad antigenic spectrum stimulate cytolytic T cells and offer increased protection against EBV infection ex vivo and in mice
Source: PLoS Pathog. 2018 Dec 6;14(12):e1007464. doi: 10.1371/journal.ppat.1007464 (PMC6298685; doi:10.1371/journal.ppat.1007464)

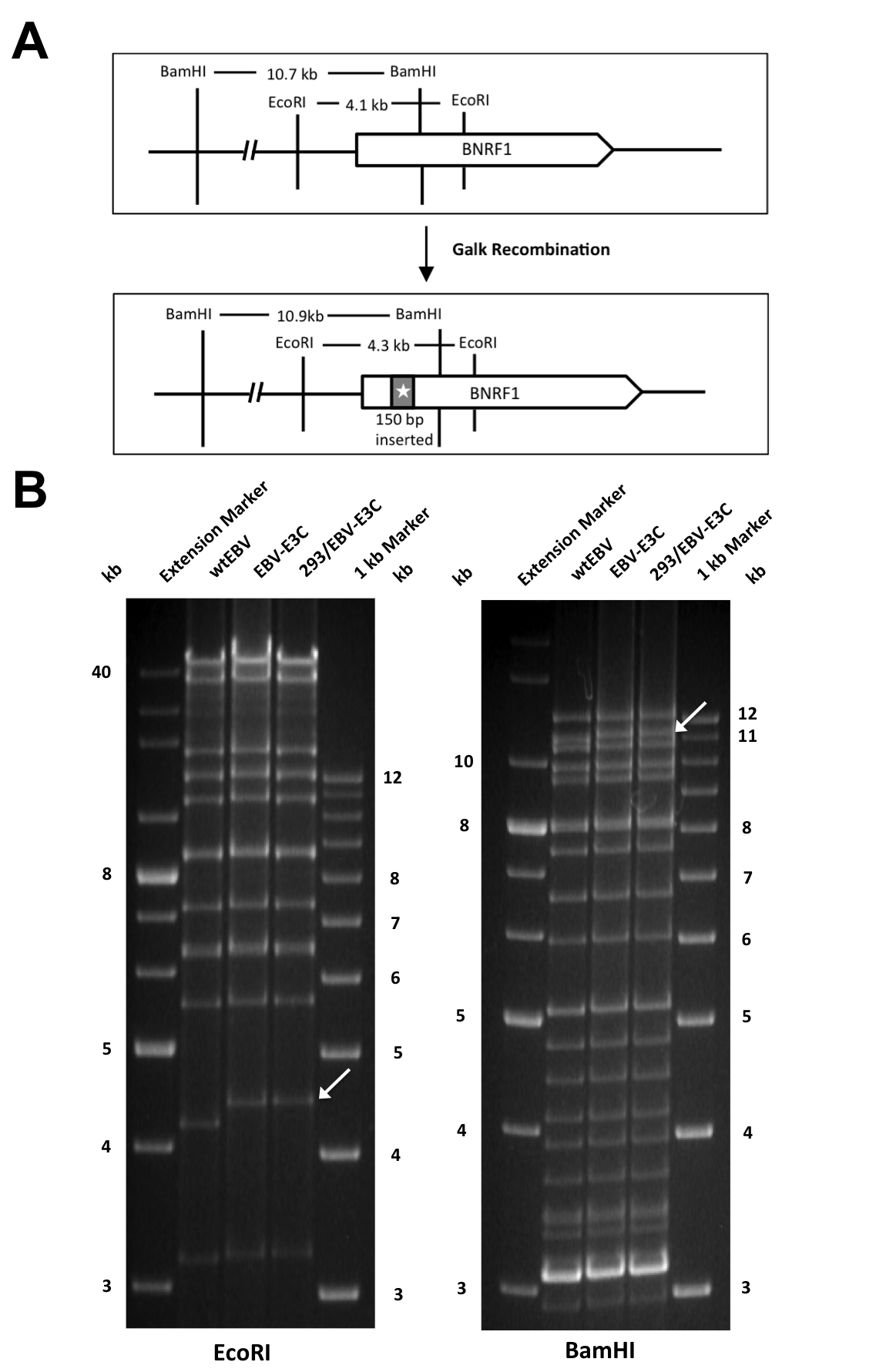

Supplement: S1 Fig — (A) Galk recombination was carried out with a 150 bp fragment corresponding to 320–344 aa and 633–656 aa of EBNA3C. EcoRI and BamHI restriction sites before and after recombination are shown, as are the size of fragments generated by these enzymes. (B) Restriction digestion with EcoRI and BamHI confirmed that EBV-E3C BAC DNA from 293 producer cells generated the same restriction fragments as EBV-E3C BAC DNA constructed in E.coli. White arrows emphasize DNA fragments that are different between wtEBV DNA (B95-8) and DNA modified with galK recombination. (TIF) [file ppat.1007464.s001.tif]

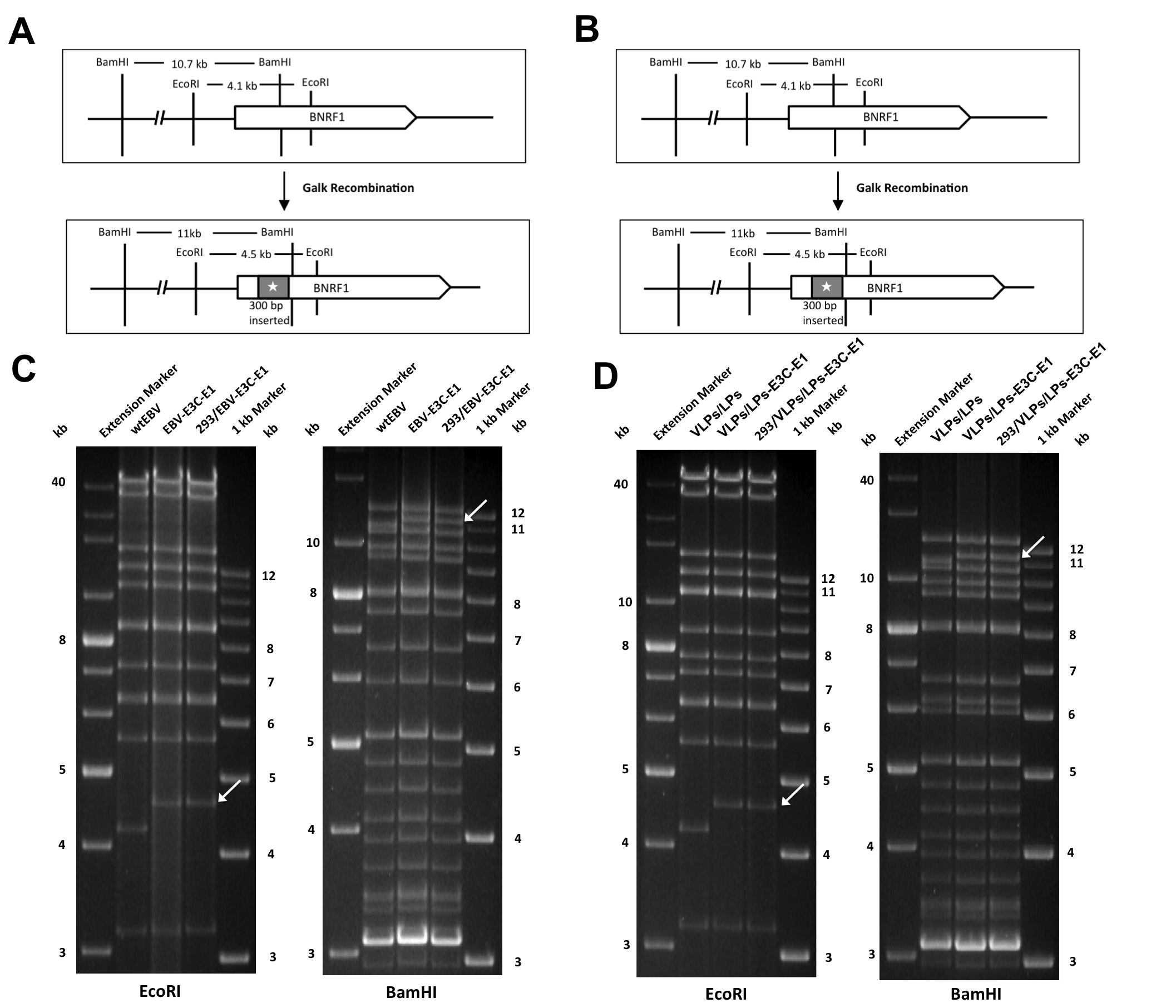

Supplement: S2 Fig — A 300 bp fragment encoding the EBNA3C (E3C) (320–344 aa and 633–656 aa) and EBNA1 (E1) (476–505 aa and 522–546 aa) was introduced into EBV (A) and VLP/LP (B) BAC DNA using galK recombination. EcoRI and BamHI restriction sites before and after recombination are shown, as well as the size of fragments generated by these enzymes. Restriction digestion with EcoRI and BamHI confirmed that EBV-E3C-E1 (C) and VLP/LP-E3C-E1 (D) BAC DNA from 293 producer cells generated the same restriction fragments as BAC DNA constructed in E.coli. White arrows emphasize DNA fragments that are different between unmodified EBV and VLP/LP DNA modified with galK recombination. (TIF) [file ppat.1007464.s002.tif]

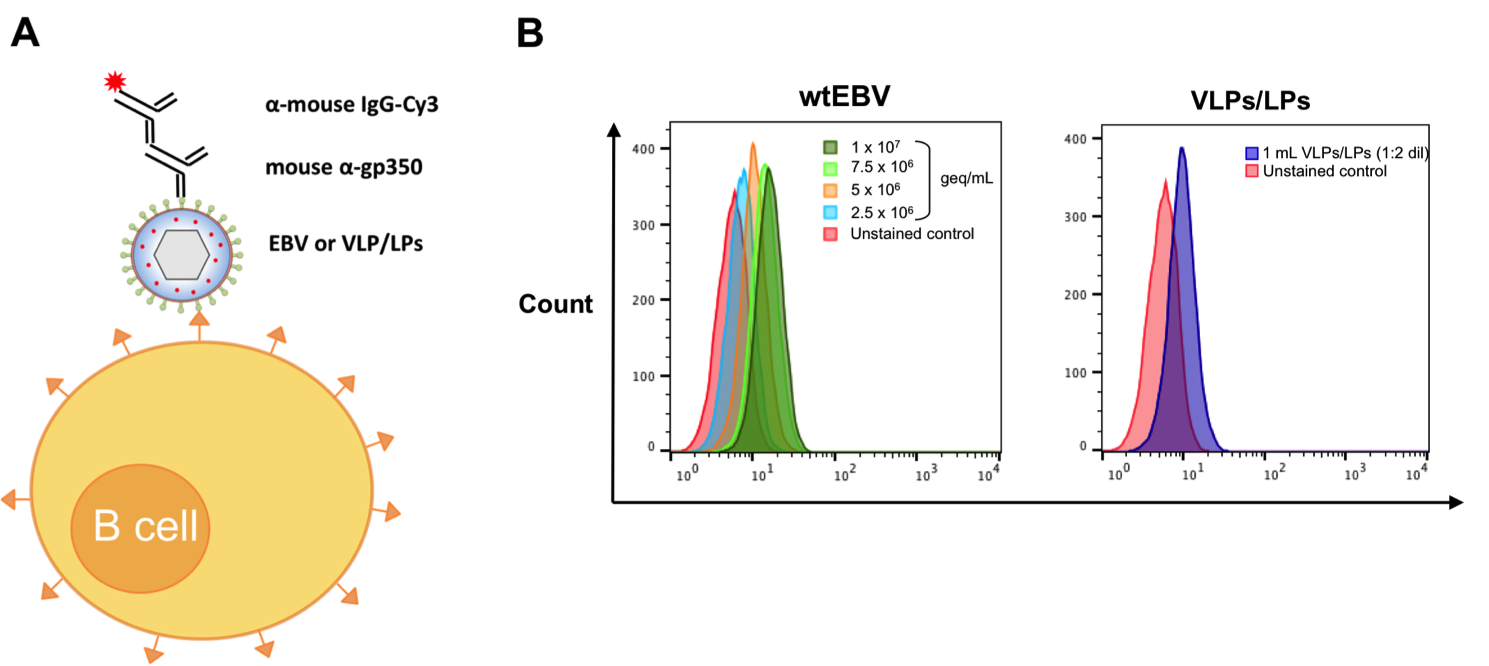

Supplement: S3 Fig — (A) The ability of VLPs/LPs and wtEBV to bind B cells was exploited for quantification purposes. B cells exposed to wtEBV or VLPs/LPs were stained with α-gp350 (72A1) and α-mouse IgG-Cy3 antibodies and analysed with flow cytometry. (B) wtEBV, previously quantified with qPCR, was added in increasing amounts (geq) to B cells and analysed with flow cytometry. This revealed a linear relationship between virus titer (geq) and median fluorescence intensity (MFI). Similarly, the MFI was determined for VLPs/LPs-E3C-E1 and the linear relationship between MFI and virus titer used for their quantification. (TIF) [file ppat.1007464.s003.tif]

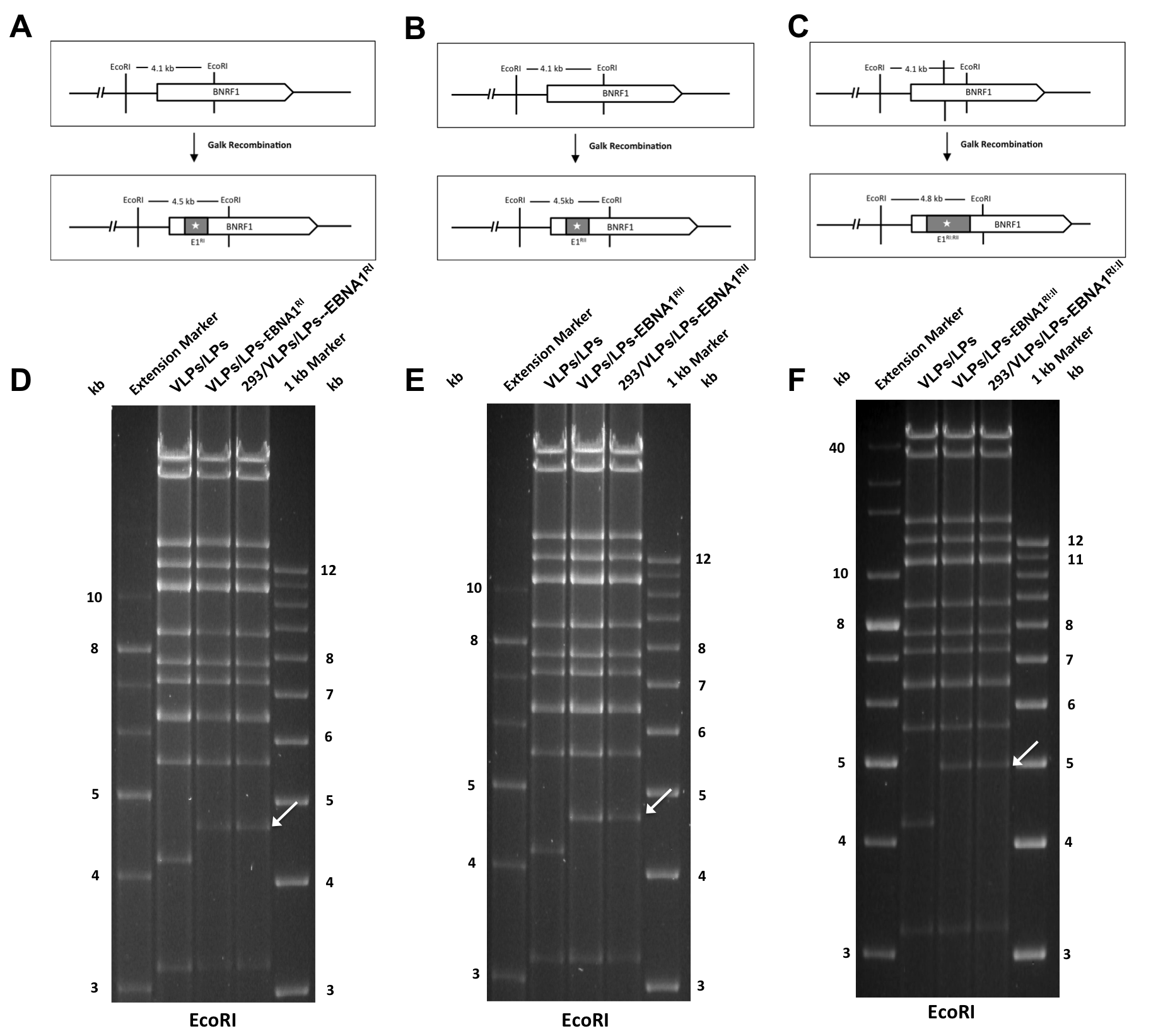

Supplement: S4 Fig — VLP/LP BAC DNA was modified with galK recombination using EBNA1-coding sequences corresponding to region I (A), II (B), and I:II (C). EcoRI restriction sites and restriction fragments are shown. Restriction digestion of BAC DNA with EcoRI confirmed that VLP/LP-EBNA1RI (D), VLP/LP-EBNA1RII (E) and VLP/LP-EBNA1RI:II (F) from producer cells was the same as BAC DNA constructed in E.coli. White arrows highlight DNA fragments that are different between unmodified VLP/LP BAC DNA and galK modified VLP/LP BAC DNA. (TIF) [file ppat.1007464.s004.tif]

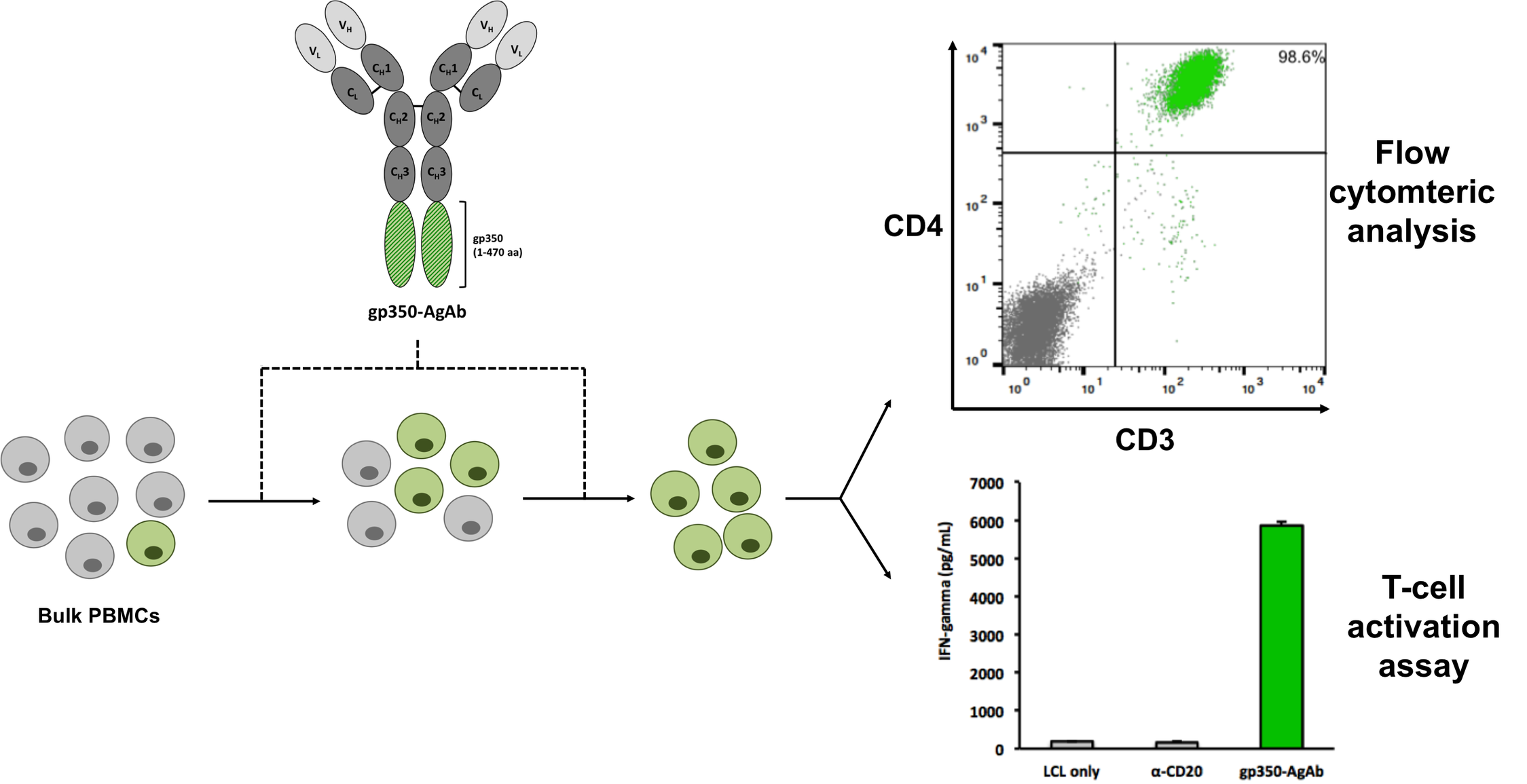

Supplement: S5 Fig — The ligand-binding domain (1–470 aa) of gp350 was fused to the CH3 domain of α-CD20, expressed in 293 cells and used to stimulate (dotted lines) the PBMCs from an unhaplotyped EBV-positive donor in the presence of IL-2. After a minimum of six stimulation cycles, ex vivo cultures were stained for CD3 and CD4 and analysed by flow cytometry. The percentage of CD3+CD4+ double-positive cells in ex vivo cultures is shown. Unstained cells are shown in grey. A T-cell activation assay was performed to confirm that the expanded T cells were specific for gp350-AgAb. Autologous LCLs were pulsed with medium, unmodified α-CD20 or gp350-AgAb and then cocultured with the CD4+ T cells. The release of IFN-γ was measured by ELISA. (TIF) [file ppat.1007464.s005.tif]

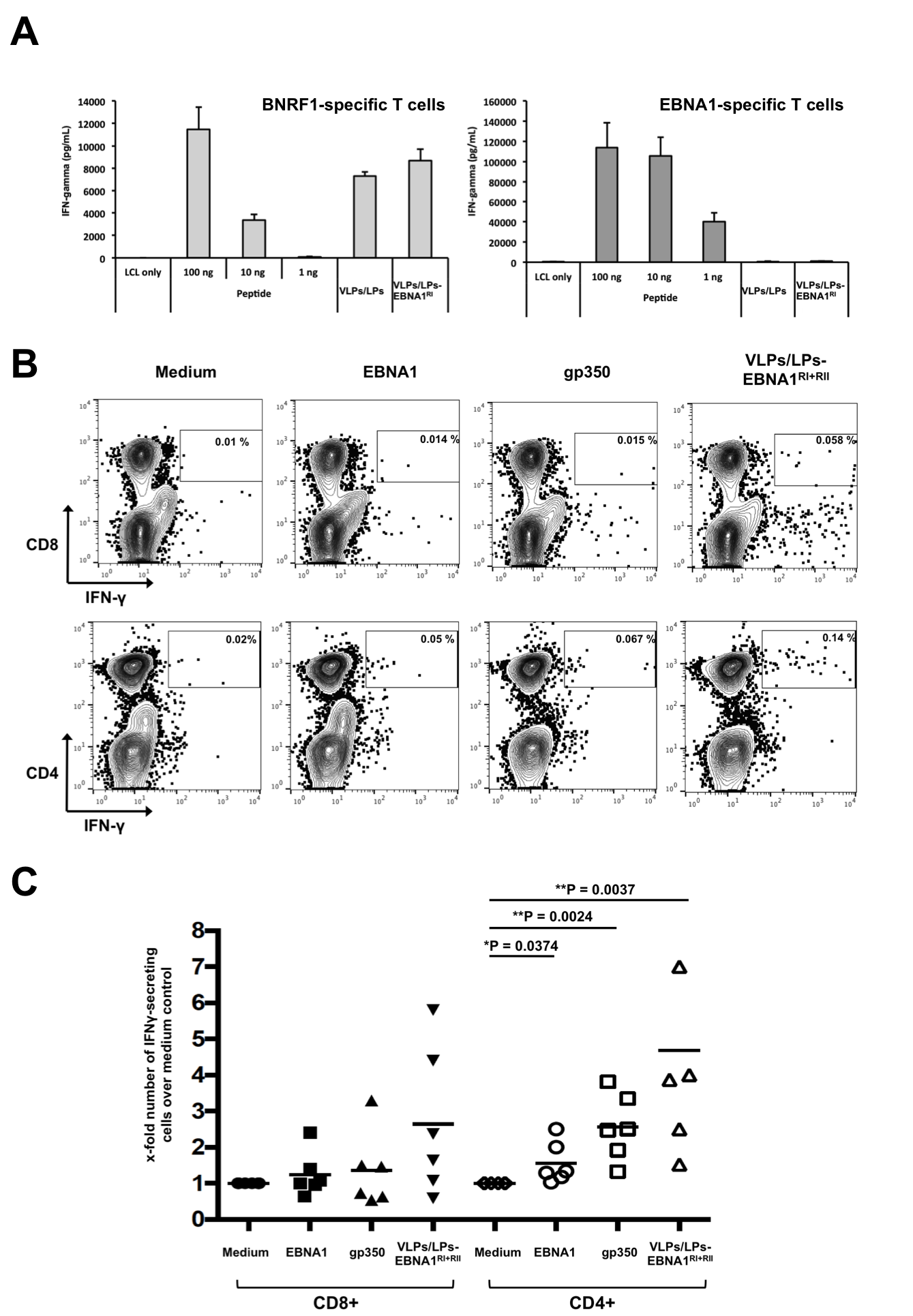

Supplement: S6 Fig — (A) Autologous LCLs were pulsed with unmodified VLPs/LPs (1 x 10^6^ particles) or VLPs/LPs-EBNA1RI (1 x 10^6^ particles) and then cocultured with T cells specific for the CD4-restricted BNRF1 VSD epitope (1006–1017 aa) or the CD8-restricted EBNA1 HPV epitope (407–417 aa). T-cell activity was determined by quantifying IFN-γ release with ELISA. The assay was performed in triplicate and standard deviations are illustrated. (B) PBMCs from EBV-positive donors were stimulated with VLPs/LPs-EBNA1RI+RII for a single round and the frequencies of IFN-γ+CD8+ (top row) and IFN-γ+CD4+ (bottom row) T cells were determined after restimulation with medium, EBNA1 peptide, gp350-AgAb and VLPs/LPs-EBNA1RI+RII. Representative data from six experiments are shown and displayed percentages are of total cells. (C) A summary of IFN-γ secretion from six donors. Statistical analysis was performed using a two-tailed student t-test. Only P values lower than 0.05 are shown. (TIF) [file ppat.1007464.s006.tif]

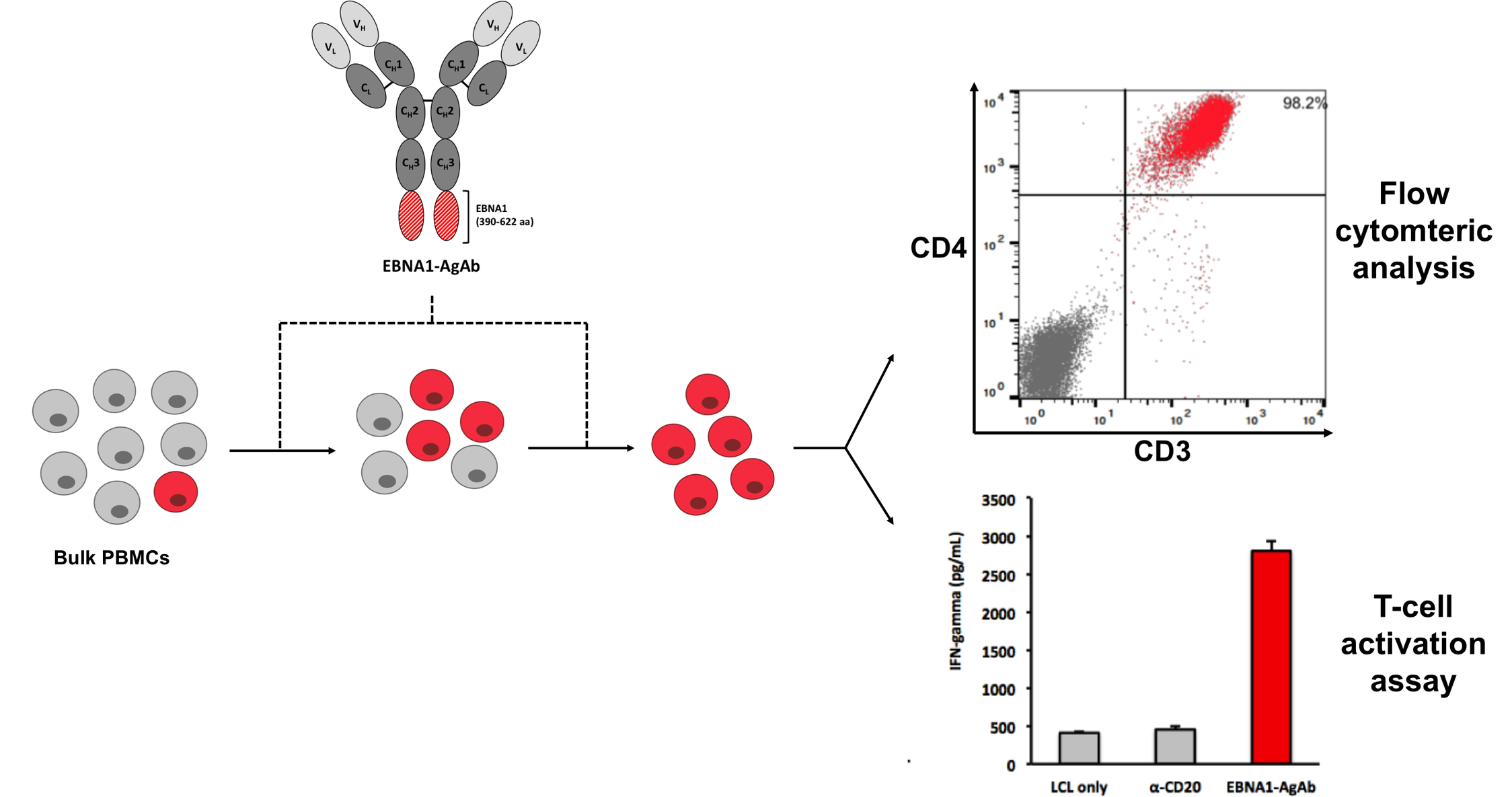

Supplement: S7 Fig — An epitope-rich region of EBNA1 (390–622 aa) was fused to the CH3 domain of α-CD20, expressed in 293 cells and used to stimulate (dotted lines) the PBMCs from an unhaplotyped EBV-positive donor in the presence of IL-2. After a minimum of six stimulation cycles, ex vivo cultures were stained for CD3 and CD4 and analysed with flow cytometry. The percentage of CD3+CD4+ double-positive cells are shown. Unstained cells are shown in grey. A T-cell activation assay was performed to confirm the specificity of the expanded T cells towards the EBNA1-AgAb. Autologous LCLs were pulsed with medium, unmodified α-CD20 or EBNA1-AgAb and then cocultured with the CD4+ T cells. The release of IFN-γ was measured by ELISA. (TIF) [file ppat.1007464.s007.tif]
