# Supplementary material for: Immunogenic particles with a broad antigenic spectrum stimulate cytolytic T cells and offer increased protection against EBV infection ex vivo and in mice
Source: PLoS Pathog. 2018 Dec 6;14(12):e1007464. doi: 10.1371/journal.ppat.1007464 (PMC6298685; doi:10.1371/journal.ppat.1007464)
Supplement: S1 Table — (PDF) [file ppat.1007464.s008.pdf]

Table S1: List of oligonucleotides

Primer	Sequence	Clone
EBNA1for	TAGCGGCCGCACAGTCATCATCATCCGGGTCTC	EBNA1-AgAb
EBNA1rev	TTTCATAGATCTTAATGGTGATGGTGATGATGCGCGGCAGCCCCTTCC	
gp350 for	CGTAGCGGCCGCAATGGAGGCAGCCTTGCTTG	gp350-AgAb
gp350 rev	TTTCATAGATCTTAATGGTGATGGTGATGATGGGTGGATACAGTGGGGCCTG	
GalKfwd	GAGCAGGGTGAACACTTGGGCACGGAGAGTGCCCTGGAGGCCTCAGGCAACCTGTTGACAATTAATCATCGGCA	EBV-galK
Galkrev	ATGTGGAAGGCCTTGCCATCCAGTCTGGTCCGTAGGCATACACATAGTTGTCAGCACTGTCCTGCTCCTT	
GalKfwd	GAGCAGGGTGAACACTTGGGCACGGAGAGTGCCCTGGAGGCCTCAGGCAACCTGTTGACAATTAATCATCGGCA	VLPs/LPs-galK
GalKrev	ATGTGGAAGGCCTTGCCATCCAGTCTGGTCCGTAGGCATACACATAGTTGTCAGCACTGTCCTGCTCCTT	
E3Cfwd	GAGCAGGGTGAACACTTGGGCACGGAGAGTGCCCTGGAGGCCTCAGGCAATGCACCACCTAATGAAAATCCATATCAC	EBV-E3C
E3Crev	ATGTGGAAGGCCTTGCCATCCAGTCTGGTCCGTAGGCATACACATAGTTGCCTGACGCAGGTTTACGGC	
E1-E3fwd	AGCAGGGTGAACACTTGGGCACGGAGAGTGCCCTGGAGGCCTCAGGCAACAATGCACCACCTAATGAAAATCC	EBV-E3C-E1
E1-E3Crev	CATGTGGAAGGCCTTGCCATCCAGTCTGGTCCGTAGGCATACACATAGTTTCCAGGGGCCATTCCAAAG	
E1-E3fwd	AGCAGGGTGAACACTTGGGCACGGAGAGTGCCCTGGAGGCCTCAGGCAACAATGCACCACCTAATGAAAATCC	VLPs/LPs-E3C-E1
E1-E3Crev	CATGTGGAAGGCCTTGCCATCCAGTCTGGTCCGTAGGCATACACATAGTTTCCAGGGGCCATTCCAAAG	
E1^{RI}fwd	TAATCCCTCAGGCCAGTCATCATCATCCGGGTCTCCAC	VLPs/LPs-EBNA1^{RI}
E1^{RI}rev	TTAGATCCTGAGGCACTACCTCCATATACGAACACACCGGC	
E1^{RII}fwd	GGCACTACCGACGAAGGAACTTGGGTCG	VLPs/LPs-EBNA1^{RII}
E1^{RII}rev	GGCCGCGGCAGCCCCTTCCAC	
E1$^{RI:II}$fwd	GGCACTACCGACGAAGGAACTTGGGTCG	VLPs/LPs-EBNA1$^{RI:RII}$
E1$^{RI:II}$rev	GGCCGCGGCAGCCCCTTCCAC	
